# Wide and Deep Imaging of Neuronal Activities by a Wearable NeuroImager Reveals Premotor Activity in the Whole Motor Cortex

**DOI:** 10.1038/s41598-019-44146-x

**Published:** 2019-06-10

**Authors:** Takuma Kobayashi, Tanvir Islam, Masaaki Sato, Masamichi Ohkura, Junichi Nakai, Yasunori Hayashi, Hitoshi Okamoto

**Affiliations:** 1 Laboratory for Neural Circuit Dynamics of Decision Making, RIKEN Center for Brain Science, Wako, Saitama 351-0198 Japan; 2 Graduate School of Science and Engineering, Saitama University, Wako, Saitama 338-8570 Japan; 3 Brain and Body System Science Institute, Saitama University, Wako, Saitama 338-8570 Japan; 4 Laboratory for Mental Biology, RIKEN Center for Brain Science, Wako, Saitama 351-0198 Japan; 5 RIKEN Center for Brain Science, Wako, Saitama 351-0198 Japan; 6 Department of Pharmacology, Kyoto University Graduate School of Medicine, Kyoto, 606-8501 Japan

**Keywords:** Motor cortex, Motion detection, Fluorescence imaging

## Abstract

Wearable technologies for functional whole brain imaging in freely moving animals would advance our understanding of cognitive processing and adaptive behavior. Fluorescence imaging can visualize the activity of individual neurons in real time, but conventional microscopes have limited sample coverage in both the width and depth of view. Here we developed a novel head-mounted laser camera (HLC) with macro and deep-focus lenses that enable fluorescence imaging at cellular resolution for comprehensive imaging in mice expressing a layer- and cell type-specific calcium probe. We visualized orientation selectivity in individual excitatory neurons across the whole visual cortex of one hemisphere, and cell assembly expressing the premotor activity that precedes voluntary movement across the motor cortex of both hemispheres. Including options for multiplex and wireless interfaces, our wearable, wide- and deep-imaging HLC technology could enable simple and economical mapping of neuronal populations underlying cognition and behavior.

## Introduction

Wearable imaging instruments represent an emerging class of powerful and versatile measurement tools for *in vivo* functional analysis of the brain in freely moving animals^1–4^. Wearable microscopes such as head-mounted 2-photon microscopes, miniature endoscopes, fiber photometers, and other implantable devices have already made significant contributions in neuroscience^5–9^. Many of these instruments also incorporate recent improvements in fluorescent probe technology, such as the genetically encoded Ca^2+^ indicators^10–13^, which enable long-period, real-time imaging of neuronal activity at high signal-to-noise ratios in the living animal brain. Despite such advances, wide-field imaging of cortico-cortical inter-regional interactions at high spatiotemporal resolution remains difficult. Most current instruments only allow the capture of images from a single or limited number of focal planes. To address these problems, we developed a novel wearable fluorescence imaging system containing a head-mounted laser camera (HLC) with deep-focus and macro photographic lenses that can comprehensively analyze neuronal activity in the mouse cerebral cortex.

Traditionally, researchers have attempted to improve microscopic optics to obtain wider and deeper fields of view under the restricting design conditions of a defined focal plane and optical aberration correction. Here, we tried to achieve the same goal, but with an alternative approach using a deep-focus optical system that can integrate images of objects at different depths and perspectives into a single-plane image. This apparatus adopts an optical system similar to the one used in inexpensive compact cameras and smart cellular phones. Many types of camera modules are commercially available, thus we could manufacture a compact HLC imaging system on a purpose-built or mass-production commercial scale, and at a low cost of less than $ 1500 as raw material costs.

In this study, we used a hand-made HLC for *in vitro* and *in vivo* fluorescence Ca^2+^ imaging to assess whether physiological neuronal activity could be visualized over a wide view in the deep cortical layers of a freely moving mouse. We also show that we can resolve the information of activities of individual cells by application of proper image processing algorithm. First, we imaged individual neurons with orientation selectivity in the visual cortex to analyze cellular physiology, and second, we applied the HLC to identify the cellular assembly used for premotor activity during the planning phase before the initiation of voluntary movement in the motor cortex of both hemispheres.

## Results

### Development of a wearable instrument for fluorescence imaging of neural activity in the cerebral cortex

**Figure 1:**
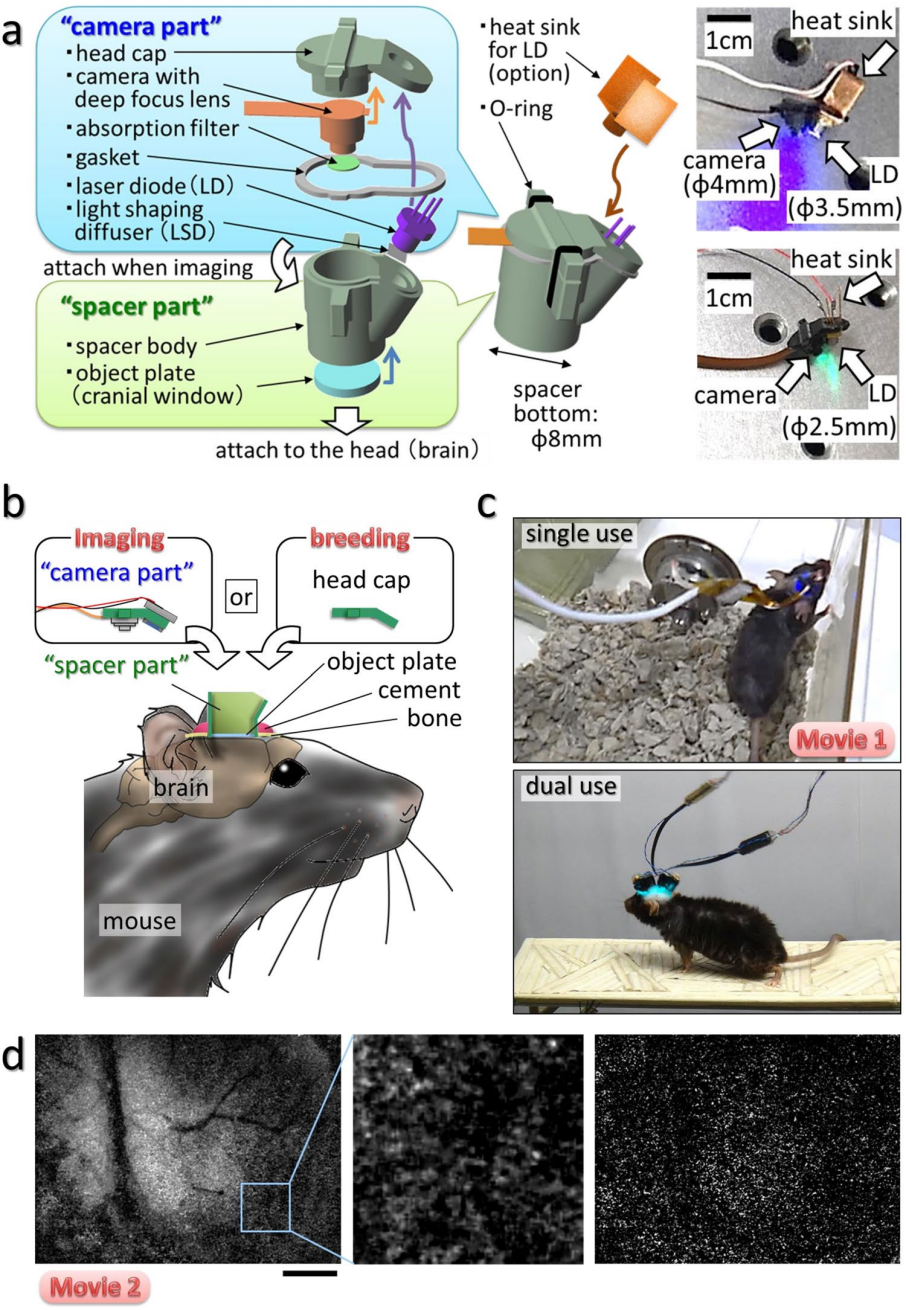
Wearable system for imaging the cerebral cortex in freely moving animals. (**a**) Schematic image of the head-mounted laser camera (HLC) is shown (see also Supplementary Fig. 1). Right photos show the HLCs for green (GFP) or red fluorescence (RFP) imaging. (**b**) Schematic image of the HLC imaging system. The camera part is attached to the spacer part when imaging is performed, and the head cap is attached to the spacer part when the mouse is housing. (**c**) Freely moving mice equipped with a single or dual HLC’s are shown in the right column images (Supplementary Movie 1) (see details of surgical operation in Supplementary Fig. 2). (**d**) Fluorescence Ca^2+^ imaging of the occipital cortical area including the visual cortex on an awake CaMK2a-G-CaMP mouse using the HLC (see also Supplementary Movie 2). The left image is a representative single frame of the movie. The middle image is a magnified view of the inset in the left image. The right image is a representative frame of the subtracted imaging movie, which was made by subtracting the average of the FI of each pixel from the FI of the same pixel during the first 10 frames of the imaging movie. The HLC can visualize excitatory neuronal activity as blinking light spots, and also visualize the spatial distribution of fine capillaries with a wide field of view (4.25 × 5.66 mm). The view area of the HLC is adjustable. Bar indicates 1 mm.

Figure 1 demonstrates our wearable fluorescence imaging system developed to visualize neural activity in the cerebral cortex of freely moving mice (Fig. 1a–c, Supplementary Fig. 1, Methods section; surgical method is explained in Supplementary Fig. 2a). The wearable apparatus consists of a separable camera and a spacer with a cranial window at its base. Imaging of the cortex is performed by the camera component through the cranial window. The object plate of the cranial window makes direct contact with the surface of the cortex (Fig. 1b). Repeated imaging of a specific brain area can be easily carried out while housing the mouse for a long period (Supplementary Fig. 2b). By equipping a suitable excitation light source and an absorption filter, the HLC can perform imaging for green or red fluorescence (right panels in Fig. 1a). The HLC is compact and lightweight, and therefore does not impede an animal’s normal behavior (Supplementary Fig. 1c, Supplementary Fig. 3d,e, and Supplementary Movie 1). We confirmed that the imaging of neuronal activities by the HLC attached to the head of freely moving mice causes no significant increase of stress, and does not affect the locomotor activity and the behavioral pattern (Supplementary Fig. 3).

Additionally, multiple cameras can be attached to the heads of small mice such as the C57BL/6 line (Fig. 1c, Supplementary Fig. 2c–e) to enable the simultaneous imaging of multiple loci, which previously has proved difficult. Importantly, since the wearable HLC is robustly fixed to the mouse skull, normal body movements do not perturb the wide-view fluorescence imaging (Fig. 1d, Supplementary Movie 2). The number of blinking spots representing presumptive cellular elements in the field of view in Fig. 1d was estimated at approximately 10,000 (Supplementary Fig. 4a,b, Supplementary Movie 3).

We used a laser diode (LD) in the HLC as an excitation light source. Thus, to prevent possible temperature increases in the diode due to continuous lighting during long-term imaging, we tested an LD driver with an attached pulse generator to toggle the LD on and off (Supplementary Fig. 5a) and heat sinks of different sizes. This modified design seemed to effectively suppress temperature changes in the LD that could adversely affect the neuronal imaging (Supplementary Fig. 5b–f). Based on the results, type #1 heat sink was mainly used for *in vivo* imaging in this paper. Furthermore, pulse driving strengthens the laser light, and such an integrated light source makes the system more adaptable to wireless control than one dependent on an external light source via optical fiber (Supplementary Fig. 6). Therefore, the HLC imaging system is suitable for use on constantly moving animals by using either wired or wireless transmission because the number of output channels is small enough to allow a USB connection and low electric power consumption with a CMOS image sensor.

**Figure 2:**
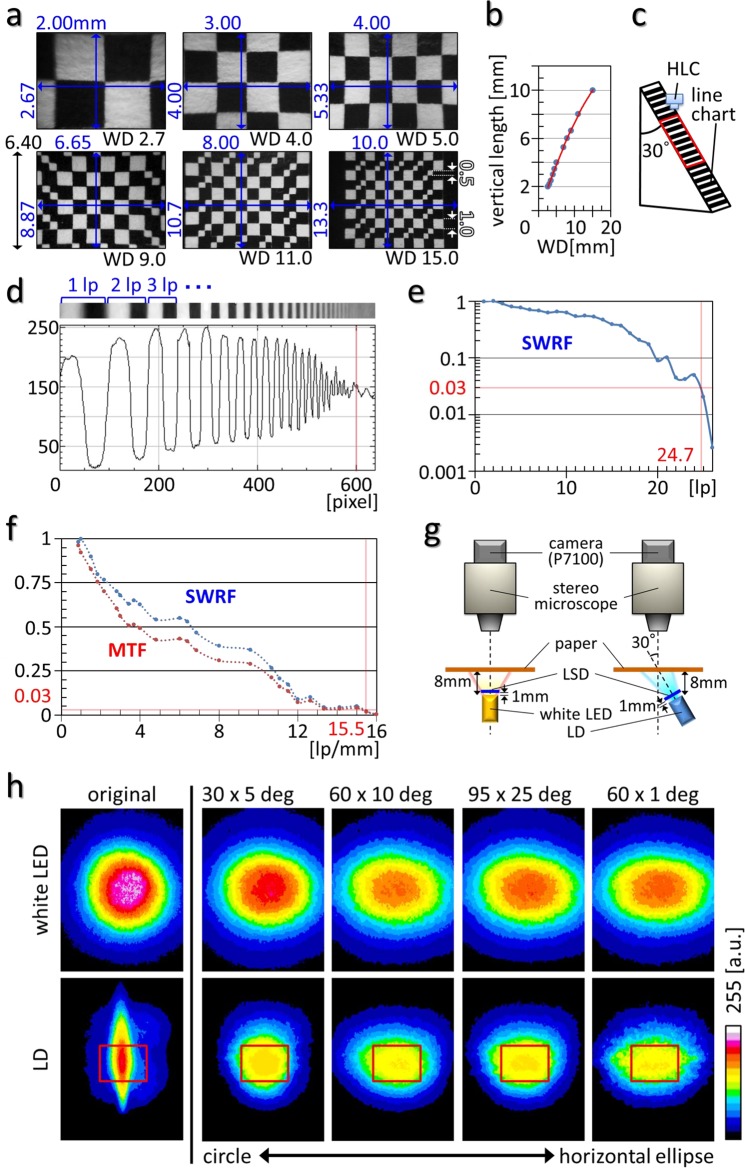
Optical specifications and adjustments of the HLC. (**a**,**b**) A checkerboard-design chart was captured by the HLC using different fields of view. The size of a small or large square is 0.5 or 1.0 mm. All results are shown in (**b**), using the representative images shown in order of size of view from (**a**). The red line in (**b**) indicates an approximate curve, expressed by Y = −1.62 × 10^−2^X^2^ + 9.4 × −0.48, and the correlation coefficient is R^2^ = 0.999. The units of numbers are all [mm], WD = working distance. (**c**) Apparatus used for measuring a line-spread function (LSF) of the HLC using a line chart. The red square indicates the view area of the HLC. (**d**) The upper picture shows the image of the line chart taken by the HLC, and the lower graph indicates the LSF (ordinate, light intensity; abscissa, vertical position of the image). The red line corresponds to the position of the red lines in (**e**,**f**). (**e**,**f**) A square wave response function (SWRF) was calculated based on the LSF (**e**) (lp = line pair, for a detailed calculation process see Methods section). A modulation transfer function (MTF) was calculated based on the SWRF (**f**). The horizontal and vertical red lines were drawn to cross at the point where the SWRF = 0.03. (**g**) Schematic image representing the light shape measurements. Several types of light-shaping diffusers (LSDs) were attached in front of the white LED or LD at a distance of 1 mm. (**h**) The distributions of illumination by an LD, whose original beam divergence was 3.3 × 14 deg at 40 mA, are shown as an 8-bit pseudo-colored image. The distributions of illumination by a white LED are shown as controls. Numbers at the top of the images show the LSD characteristics. Images are arranged according to the order of deviation of the LSD from the circle to the horizontal ellipse. Red squares indicate the typical view area of the HLC (4.0 × 5.3 mm).

Before moving to *in vivo* imaging, the *in vitro* optical specifications of the HLC were investigated (Fig. 2a–f, Methods section). A checkerboard chart (Fig. 2a,b) or a line chart (Fig. 2c–f) was captured by the HLC, and each optical parameter was calculated based on the actual measurement values. As a result, a maximum spatial resolution 4.17 µm/pixel was obtained if the blurring of edges in the images were ignored (Fig. 2a). The optical distortion was 2.78% at maximum (television distortion = 3.91%), and the depth of field exceeded 19.5 mm (see Methods section for details of the calculations).

Next, the optical processing conditions for homogenizing the flat irradiation shape of a coherent laser beam emitted by the LD were examined using a light-shaping diffuser (LSD) (Fig. 2g,h, Supplementary Fig. 5g,h). Based on actual measurements using various types of LSD (Fig. 2h) and comparison with the theoretical value with simulation (Supplementary Fig. 5g,h), a 60 × 10 degree LSD was found to be the most preferable. Accordingly, we expected that the HLC equipped with a deep-focus optical system could capture images from wide brain areas and various depths at quasi-cellular resolution.

**Figure 3:**
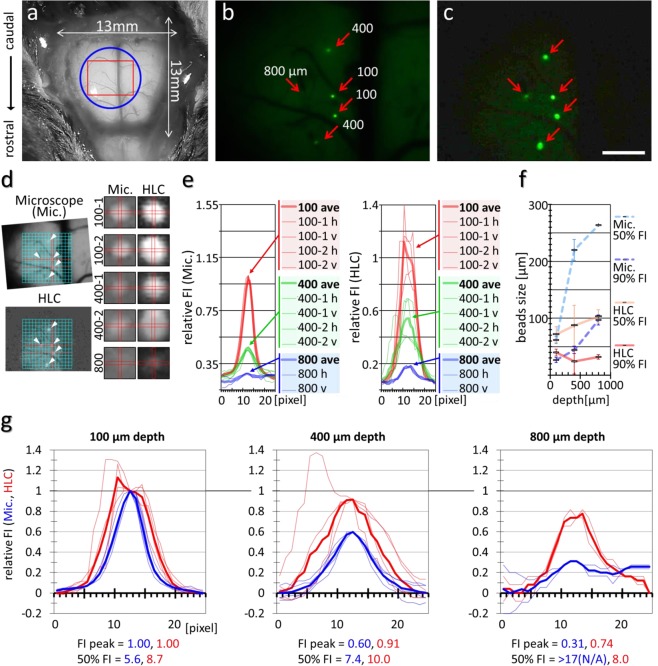
The HLC can acquire the fluorescence signal of the beads implanted into the deep layers of the cerebral cortex. (**a**) Fluorescent beads were implanted into the mouse cortex after a craniotomy. The blue circle and red square indicate the spacer position and the imaging area of the HLC, respectively. (**b**,**c**) Fluorescence images taken by a fluorescence stereo microscope (**b**) and the HLC (**c**). Red arrows indicate the positions of implanted 15-µm beads, and the numbers indicate the depth of implantation. Bar indicates 1 mm. (**d**) In the two left images, the size and position of (**b**,**c**) were aligned based on the pattern of blood vessels and the central position of the beads implanted at a depth of 100 µm (red lines). White arrowheads indicate the bead positions. The right two rows of 10 images show the magnified images of each bead taken by the stereo microscope (Mic., left column) or the HLC (right column). The vertical (v) and horizontal (h) rectangles were drawn to cross at the center of each bead. (**e**) Fluorescence intensity (FI) distribution along the (v) and (h) in (**d**) were measured, and the relative FIs against averaged FI at the center of the bead at a 100-µm depth are shown. (**f**) A summary of (**e**) is shown. The image size of the fluorescent beads was calculated according to the number of pixels in the image. By comparing the FI at the centers and perimeters of each light spot, the number of pixels of >50% FI and >90% FI were counted. In the graph, error bars indicate the standard deviation. Note that the values >90% of the HLC stay almost invariant regardless of depth (red line). (**g**) Normalized relative FI of each depth of the fluorescent beads are individually shown. Considering the relative FI at a depth of 100 µm as a standard, the maximum value or the minimum value of relative FI were calculated as 1 or 0. The blue or red number in each graph indicates the number of pixels of 50% FI (FWHM, the full width half max) of the stereo microscope (Mic.) or the HLC. N/A = not available.

To verify the imaging ability of the HLC, *in vitro* and *in vivo* fluorescence-imaging tests were performed. First, images of the fluorescent beads implanted into the cerebral cortex of the mouse were compared between the HLC and a conventional stereomicroscope (Fig. 3a–c). The HLC, but not the conventional stereomicroscope could detect beads of similar size to cells in the deep cortex even at 800 µm in depth (Fig. 3d–g). Additionally, the deep-position HLC images showed less blurring of the bead shapes than those taken by stereomicroscope (Fig. 3e–g). The findings indicate that the HLC can acquire fluorescence signals deep in the cerebral cortex compared to a conventional wide-field fluorescence microscope. Although it would not be so easy to attribute this difference in the capacity to detect the signals from deeper tissues between the HLC and the conventional stereomicroscope to a single cause because of the difference in the intensity of the excitation light, we think that the difference in the focal depth between these microscopes is important, *i*.*e*. HLC with pan-focal optics *v*.*s*. the conventional stereomicroscope with a focal depth of 500 µm above and below the best focal point. Accordingly, the fluorescent beads located 800 µm in depth were out of focus in the imaging by the conventional stereomicroscope as we put the focus upon the beads nearest to the surface (100 µm in depth).

**Figure 4:**
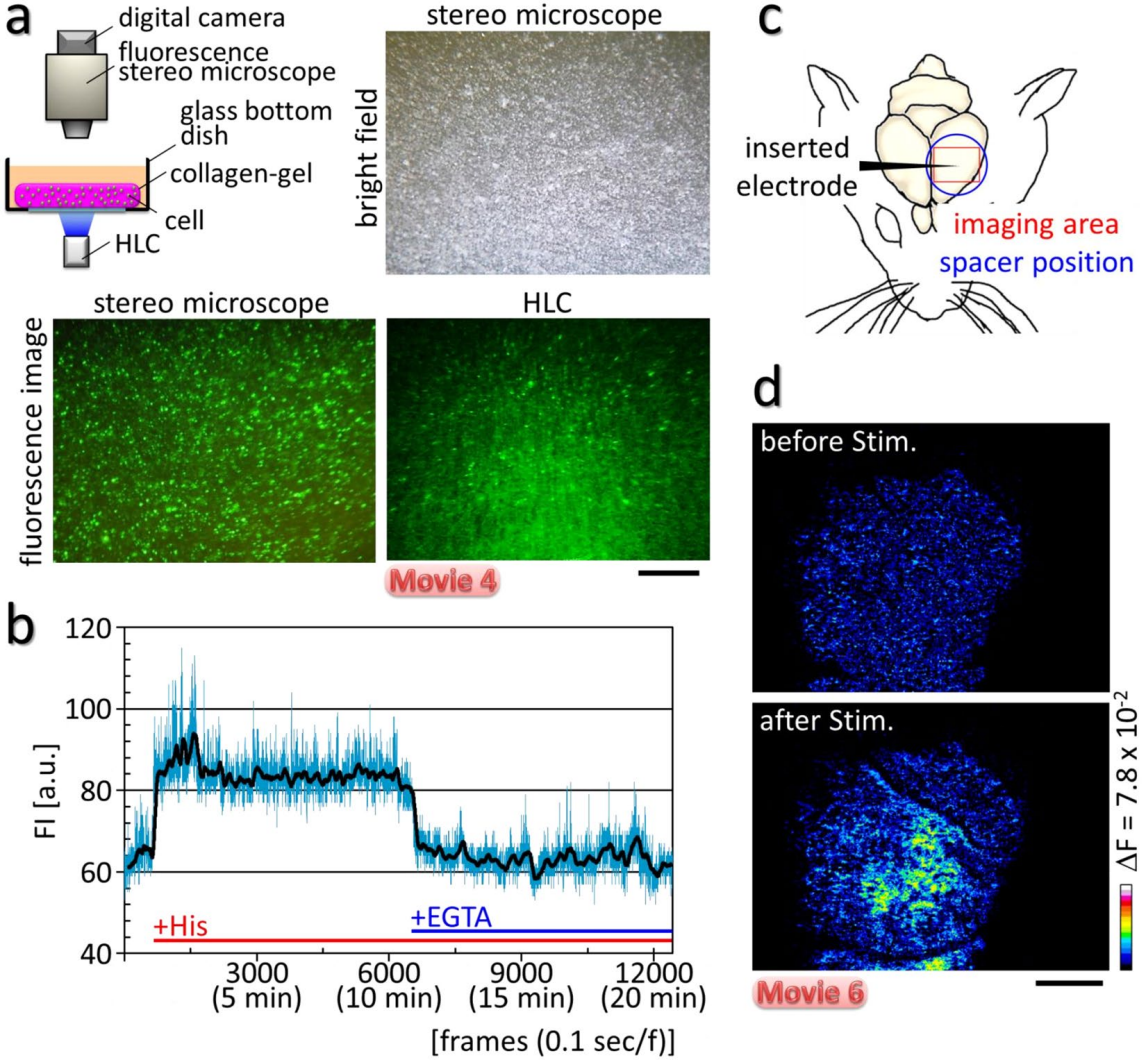
The HLC visualized intracellular Ca^2+^ dynamics of individual cells in a 3D culture, and evoked neuronal activity in the deep layers of cerebral cortex. (**a**) Schematic image of the *in vitro* experiment. The sheet of 3D cultured Hela cells transfected with the G-CaMP6 gene was placed into a glass bottom dish, and Ca^2+^ imaging was performed with the HLC. Brightfield and fluorescence images were taken by a stereomicroscope or the HLC. The transfected cells emitted green fluorescence and were distributed sparsely across various depths. Bar indicates 1 mm. (**b**) The graph shows the changes in FI of a single cell. The FI increased after histamine administration (+His), and then decreased when a solution of EGTA was applied. The black line indicates the mean FI for each of 5 frames. (**c**) Schematic diagram for the *in vivo* experiment. An electrode was inserted into the cortex of a Thy1-G-CaMP7 mouse from the side after craniotomy. Then, the HLC was applied to the cortex and Ca^2+^ imaging was performed. (**d**) Pseudo-color images are shown for the change in FI before and after electrical tetanic stimulation that was applied by an inserted electrode (see Supplementary Fig. 7c for more detail). Bar indicates 1 mm.

Second, the Ca^2+^ imaging was performed with 3D cultured cells made by embedding transfected Hela cells in an extracellular matrix gel as a mock brain tissue (Fig. 4a). The change ratios (ΔF/F) of the fluorescence intensity (FI) of individual cells at various depths increased with histamine administration, whereas they were decreased by EGTA administration (Fig. 4b, Supplementary Fig. 7a, Supplementary Movie 4). The results indicate that the Ca^2+^ imaging of individual cells could be performed in a 3D cell culture using the HLC.

**Figure 5:**
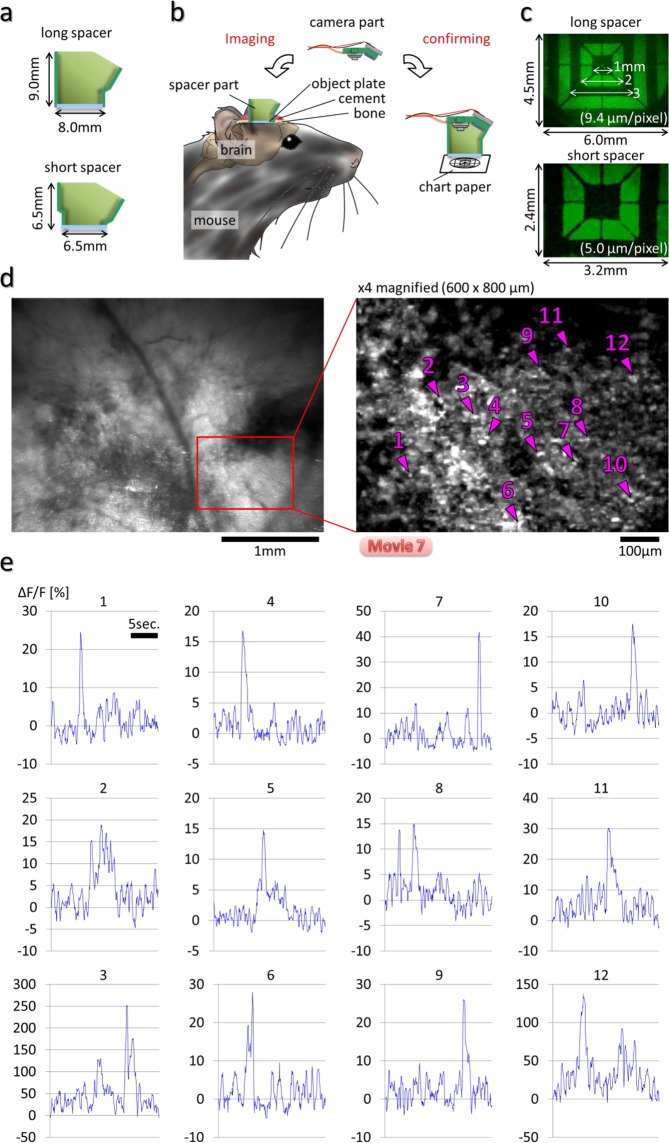
Zoom-up Ca^2+^ Imaging by using the HLC with short spacer in the freely moving mice The field-of-view size by the HLC can be changed for zooming up by twisting the lens barrel. For small field of view with short working distance after zooming up, it is preferable to use short spacer apparatus. (**a**) Schematic images of different type of spacer are shown. The spacer part can be manufactured arbitrarily by changing its design. (**b**) After imaging, the field-of-view size was confirmed by capturing the image of the reference chart. (**c**) The images of the reference chart captured by the HLC with long or short spacer are shown. (**d**) Ca^2+^ imaging was performed at the visual cortex of the CaMK2a-G-CaMP7 mouse by the HLC with the short spacer under the freely moving condition. The maximized fluorescence image of 20 fps movie for 5 min is shown at the left panel, and its magnified, maximized fluorescence image of the inset part of the left panel for 20 sec is shown on the right panel. Magenta arrowheads and numbers indicate the ROIs of which the light spots were randomly selected. The averaged and maximized movie in each 5 frames is shown in Supplementary Movie 7. (**e**) The change rates of the fluorescence intensity (ΔF/F) at each ROIs of (**d**) are shown.

**Figure 6:**
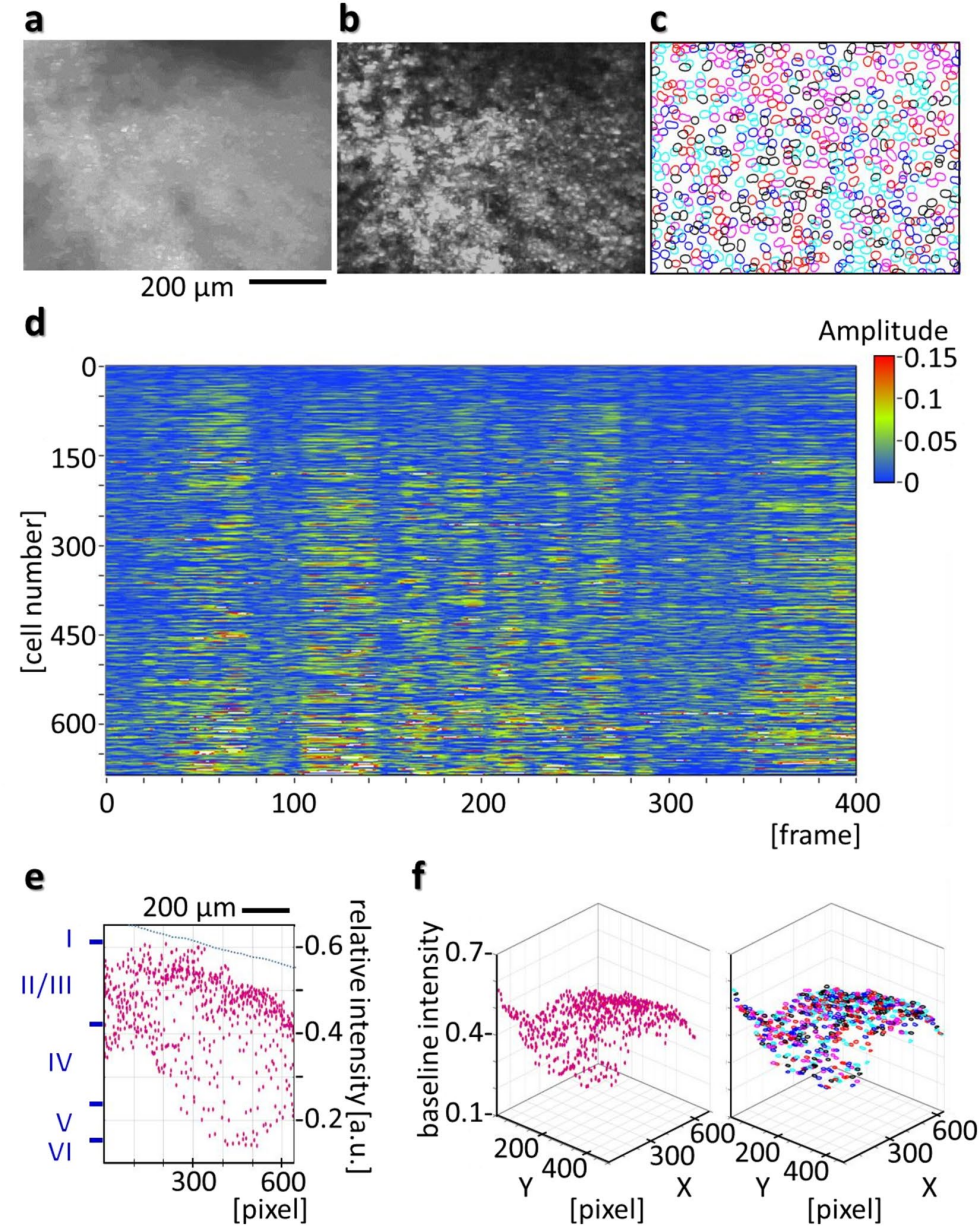
Separation and identification of superimposed signals of individual cells by NMF in the HLC image. (**a**) Ca^2+^ imaging of the CaMK2a-G-CaMP7 mouse at the visual cortex was performed under the freely moving condition. The Maximized fluorescence image at 20 fps for 20 sec at the same view point as in Fig. 5d is shown. (**b**) shows the subtracted maximized image of the movie. (**c**) Using the raw 20 fps imaging data for 20 sec (total 400 frames), it was possible to segment the image into individual ROIs (region of interest) representing cell bodies by using a NMF algorithm (Methods section). The result is shown in (**c**), which shows the boundaries of 685 detected cells, plotted with different colors. It can be noted that many cells with spatial overlap were detected. (**d**) Shows the signal extracted by NMF for each cell over time, showing spontaneous activities of cells during free moving. With the application of the NMF algorithm, baseline removal and separation of temporal signals corrupted by spatial overlap of cells was achieved. (**e**) Shows the centroid locations of the cell bodies in X-Z plane, where X is the longer image axis of (**a**) and Z is the depth of cortex corresponding to baseline value of the cell signal. Blue Roman numerals at the Z axis indicate the estimated cortical layer, assuming that deepest signal came from a depth of 1 mm. The broken blue line indicates the imaginary inclined cortical surface. (**f**) The left image shows the centroid locations of the cells in three-dimension, where X and Z axis are same as (**e**), while Y-axis corresponds to the shorter image axis of (**a**). A three dimensional distribution of the cell bodies shows the presence of cells with various depths. The right image shows the same three dimensional distributions of the cell bodies with cell boundaries showed instead of centroid.

The spacer part of the device with the cranial window is also useful to observe the cortex under a conventional or 2-photon microscope system (Supplementary Fig. 8, Supplementary Movie 5); however, a major advantage of the HLC in this regard is that a large amount of information can be obtained from a single image in real time compared to scanning multiple focal planes as needed for the same results by confocal or 2-photon laser microscopy systems. Furthermore, by using transgenic animals expressing the Ca^2+^ indicator fluorescent proteins in layer- or cell type-specific manners, we also obtained images from defined subpopulations of neurons in the brain regardless of their distribution areas. As two examples in the present study, CaMK2a-G-CaMP7 mice expressing G-CaMP specifically in excitatory neurons (Figs 5, 6, Supplementary Figs 8–10) and Thy1-G-CaMP7 mice expressing G-CaMP predominantly in layer 5/6 pyramidal neurons were imaged (Fig. 4c,d, Supplementary Fig. 7c).

For Ca^2+^ imaging of the Thy1-G-CaMP7 mouse by the HLC, we applied an electrical tetanus stimulation to the somatosensory area, and observed an increased FI around the electrode in the transgenic mouse, whereas no such evoked signals were detected in a control wild-type mouse (Fig. 4c,d, Supplementary Fig. 7c, Supplementary Movie 6). In Supplementary Movie 6, the HLC was placed on the surface of the cortex without fixing it to the cranial bone. Therefore, the image shows slight vibrations due to the muscle movement induced indirectly by stimulation of the somatosensory area, even under anesthesia. The imaging result indicates that HLC can visualize the evoked activity of cortical neurons located as deep as layer 5/6.

### The HLC can detect physiological neuronal activity at cellular resolution in the cerebral cortex including the visual area of one hemisphere in freely moving mice

**Figure 7:**
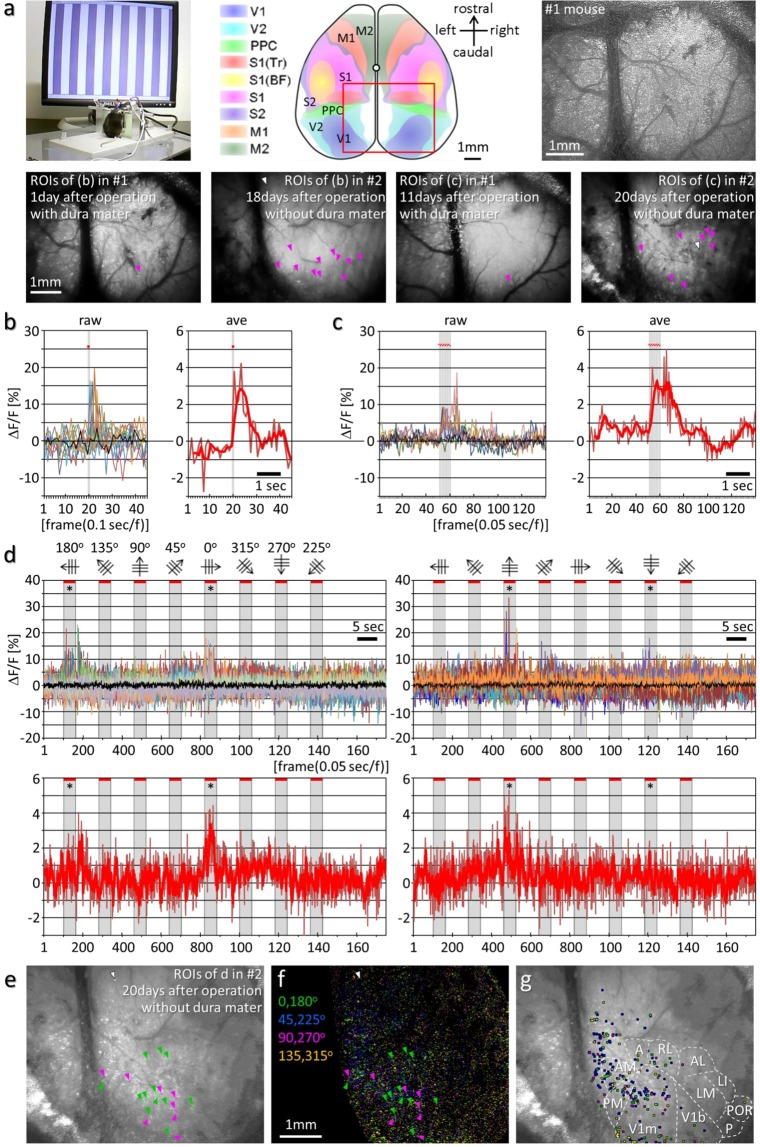
Observation of the physiological responses of individual neurons by large-scale imaging including the whole visual area of the cortex. (**a**) The upper left picture shows the experimental setup. The upper middle schematic diagram shows the HLC imaging area (red square, 4.25 × 5.66 mm) with the map of the brain area reconstructed from serial sections of the brain atlas (Supplementary Fig. 9a). Abbreviations: V1/2, primary/secondary visual cortex; PPC (PtA), posterior parietal cortex (parietal association cortex); S1 (Tr/BF)/S2, primary (trunk/olfactory barrel field)/secondary somatosensory cortex; M1/2, primary/secondary motor cortex. The upper right image is the deconvoluted fluorescence image of the occipital cortex in the awake CaMK2a-G-CaMP7 mouse taken by the HLC (see Methods section for detail). The lower four images are fluorescence images of the occipital cortex. Magenta and white arrowheads indicate the region of interests (ROIs) or negative control ROIs, and their ΔF/F are presented in (**b**,**c**). Magnified images of the lower panels are shown in Supplementary Fig. 4c,d. (**b**,**c**) The left and right graph shows the raw or averaged ΔF/F after a single (**b**) and 10x repeated flashlight stimulus (**c**), respectively, was applied by the LED. Gray vertical lines and red dots in each graph indicate the stimulation points. The red bold line data in the right graph of (**b**,**c**) indicate the means of 3 and 5 frames, respectively. Black lines mean negative control ROIs. (**d**) Drifting gratings in 8 different directions were presented. The upper and lower graphs show the raw or averaged ΔF/F. Examples of the specific ΔF/F increases according to the opposite degree stimulation (asterisks) are shown. Data indicated by the black line indicate the ROI of the negative control, and the gray shaded time windows in each graph indicate the stimulation periods. The red bold line data in the lower graphs indicate the means of 5 frames. (**e**) Fluorescence image shows the ROIs of the #2 mouse. White arrowheads indicate negative control ROIs, and green and magenta arrowheads show ROIs for left graph or right graph in (**d**). Magnified image is shown in Supplementary Fig. 4e. (**f**) shows the distribution of the ROIs of different orientation specificity by different pseudo-colors in the same area as (**e**). See Methods for details. Magnified image is shown in Supplementary Fig. 4f. (**g**) shows the selected ROIs with high level of orientation specific responses (top 3.5%) among the ROIs in the same area as (**e**). See Methods for details. These ROIs are mostly contained in the visual area of the cortex. Magnified image is shown in Supplementary Fig. 4g. Abbreviations: V1m/b, primary visual cortex monocular/binocular zone; A, anterior; AM, anteromedial; PM, posteromedial; RL, rostrolateral; AL, anterolateral; LM, lateromedial; LI, laterointermediate; POR, postrhinal; P, posterior area.

To demonstrate the validity of the HLC, physiological responses of individual neurons during visual perceptual information processing were observed by Ca^2+^ imaging in freely moving CaMK2a-G-CaMP7 mice (Figs 5–7).

By twisting the lens barrel of the camera part of the HLC, the HLC can obtain either a narrow or wide field of view arbitrarily (Fig. 2a,b, Methods section). A short spacer was used to compensate for the reduction in the working distance by optical zooming (Fig. 5a–c). Under the freely moving condition, Ca^2+^ imaging was performed on the visual cortex of the CaMK2a-G-CaMP7 mouse (Fig. 5d,e, Supplementary Movie 7). As a result, the neuronal activity of the visual cortex can be visualized when the mouse was viewing the surrounding scenery, and the vibration derived from mouse’s behavior was not observed and the view field did not drift because of the firm attachment of the HLC to the head. It can be noted that the detection of cells with partial overlap in XY plane but separated in Z axis can raise the possibility of simultaneous recording of cells from various layers of the cortex. To address this problem, we deconvoluted the signals of overlapping cells individually by using a NMF (Non-negative Matrix Factorization) algorithm^14^ (Fig. 6, Methods section). As a result, 685 cells and their activities were detected in the magnified 600 × 800 µm image (Fig. 6c,d). The HLC captures signals from cells at various depths, superimposed in one plane. Though it is not possible to exactly determine the depth of the cells from the surface with the current HLC, we can roughly assume that cells with higher baseline activities may be located on shallower depth from the surface and reconstruct the putative distribution of cells in 3D (Fig. 6e,f).

Next, to examine the differences in physiological responses to different stimuli, a 0.1-second (sec) single flashlight stimulation or 10 repeats of a 0.5-sec flashlight stimulus (light on, 25 ms; light off, 25 ms; 10 times), was applied to the mouse by using an LED positioned in front of the left eye (Methods section). Transient increases (av. 0.50 sec, SD ± 0.14) in FI were observed at 12 fluorescence spots in the primary visual cortex (V1) when a flash stimulation was applied (Fig. 7b), whereas gradual increases in the FI of 7 spots were observed over a longer period (av. 1.09 sec., SD = ±0.41) during and after repeated flash stimuli (Fig. 7c). The ΔF/F at places other than the visual area, considered to represent basal brain activity irrelevant to visual information processing, showed a fluctuation within 3% maximal FI (black line in the graph). No significant increases of the ΔF/F at these places (black line) during the light stimulus mean that no external light was incident on the imaging area and supports the correctness of the experimental results. Evidently, the transient, significant increases of fluorescence in the visual cortex were caused by neuronal activity, and such increases were strong and long depending on the stimulation time. These results demonstrate that the HLC can differentiate the neuronal responses to different stimuli.

The complex cells in the primary visual cortex in cats show specific orientation selectivity in response to the specific directions of scanning light stimuli^15^. In rodents, the orientation selectivity is represented mainly in layer 2/3 neurons, although minor responses are also observed in the geniculate afferent fibers^16,17^. The neurons with orientation selectivity are also randomly distributed in the visual cortex of rodents, thus we examined whether the HLC could detect such neuronal activity within the visual cortex of mice.

An 8-direction drifting grating was presented to the mice, and Ca^2+^ imaging was performed (Fig. 7a, Methods section). A transient, strong increase in FI was detected at the specific light spots in V1 only when a specifically orientated grating was presented (Fig. 7d). The left column graphs indicate the examples of the 15 light spots that responded robustly to 0 and 180-degree stripes (asterisks), and did not respond at all to 90 and 270 degrees. In contrast the right column graphs indicate the examples of the 12 light spots that responded robustly to 90- and 270-degree stripes (asterisks), while none of them responded to 0 and 180 degrees. We further analyzed the orientation specificity of all ROIs of the observed area (Fig. 7e–g, see Methods for details). The responding light spots could therefore represent neurons with specific orientation selectivity. Thus, the HLC enables a broad-volume analysis of the physiological activity of single neurons in the cerebral cortex.

### Functional neuronal imaging of the whole motor cortical area reveals specific patterns of premotor activity representing discrete neuronal assemblies

We next explored the advantages of the wearable instrument for the analysis of brain activity in freely behaving subjects, including movement. For this purpose, the entire motor cortex area of a mouse was exposed on the surface of the brain, such that the HLC could visualize the entire area in both hemispheres simultaneously. Ca^2+^ imaging of CaMK2a-G-CaMP mice was performed using the HLC in the frontal cortical area including the motor cortex of freely moving animals (Supplementary Fig. 9). Different patterns of neuronal activity were elicited depending on different external stimulations of the acoustic and somatosensory modalities. Among those event-related responses, synchronized and slow wavelike activities were observed across broad areas while the mouse was standing still after the stimulation (Supplementary Movie 8).

The pre-motor readiness potential (RP) originally reported as a bereitschaftspotential, is a neuronal premotor activity elicited in the motor cortical area before the initiation of voluntary muscle movement^18^. The RP thought to occur in the higher motor cortex earlier than nerve activity to move the muscle is presumed to be the reflection of the animal’s intention to move the body^19,20^. The premotor activity for an unintended action is categorized as a Type-II RP (no preplanning) while the larger premotor activity for an intended action is distinguished as the Type-I RP (preplanned act); however, it is difficult to analyze premotor activity induced by the random behavior of animals, and the observation of a part of the motor cortex alone cannot reveal the entirety of this premotor activity. Therefore, in the present study, we attempted to measure neuronal activity in the whole motor cortical area of the mouse during voluntary movement to verify that a specific cell assembly expressing the readiness potential might exist.

**Figure 8:**
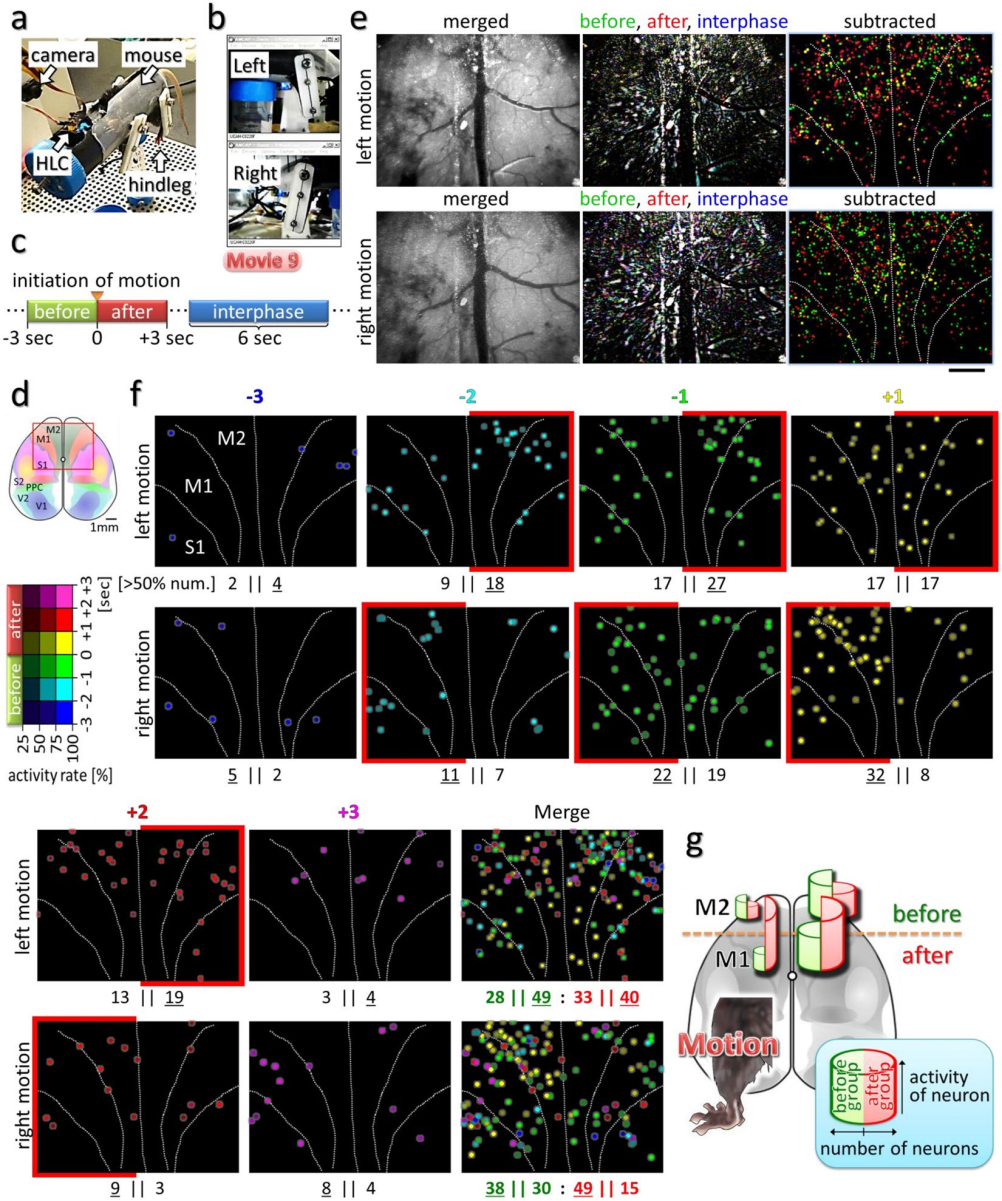
Imaging of the entire motor cortex reveals premotor activity. (**a**) The whole view of the restricted-motion experiment apparatus for mice. (**b**) Hindleg movements (kicks) of the mouse were captured by web cameras from both lateral sides of the apparatus. (**c**) Schematic diagram showing classification of the periods during the experiment. The movement state was subdivided into 3-sec durations representing “Before” and “After” the initiation of motion. “Interphase” indicates the 6-sec static state. (**d**) Schematic image showing the HLC imaging position (red square). The Ca^2+^ imaging was performed in awake CaMK2a-G-CaMP7 mice. (**e**) The upper or lower row images show the results of the Ca^2+^ imaging with left or right hindleg kicking, respectively. The left column images represent the fluorescence images in which all raw-imaging data frames in 11 or 10-time left or right kicks during the 6-sec motion state were merged into one frame by maximization. Similarly, imaging data of the “interphase” state in 3-time left or right kicks were merged, and the results are shown in the middle column by blue pseudo-coloring. Also, “before” and “after” states are shown in green and red, respectively. The right column images show the subtracted results, in which green and red represent the [“before” – “interphase”] and [“after” – “interphase”] of the middle column images, respectively. To make visual understanding easier, the light spots of the subtracted images were enlarged and made brighter. (**f**) The subtracted results of (**e**) for the spots with ΔF/F > 5% were plotted spatiotemporally for each left or right kick (Supplementary Fig. 10c). The brightness of each pseudo-colored dot represents an averaged frequency of appearance of the peak in each trial. Total number of the spots that have >50% activity frequency on the left or right hemisphere is shown below each panel. Underline means superior one at left or right. Below the panel of merge, total number from −3 to −1 or +1 to +3 is represented in green or red. (**g**) A schematic diagram summarizing the results in Fig. 8 and Supplementary Fig. 10 for the left hindleg movement. The width and height of the half columns in each hemisphere represent the number and the averaged activity of the “before-” and “after-group” defined in Supplementary Fig. 10d–g. The before-group neurons, which have a peak excitation frequency before the initiation of movement, continue to show lateralized distribution and activity both before and after the motion, while the after-group neurons show less lateralization.

We developed what we term “restriction motion experiment” to easily detect the activity associated with a specific movement (Fig. 8, Methods section). In this study, a mouse with the HLC on its head was restrained in a plastic tube with its four legs attached to splints set up for monitoring leg movements (Fig. 8a,b). Brain activity preceding the actual execution of exercise, *i*.*e*., the readiness potential, is proposed to occur in the motor cortical area^18^, thus we sought to confirm this observation using the HLC on our mouse subjects. Using the movement of a lever as an index, the neuronal activities in bilateral motor cortices were extracted immediately before and after the onset of a specific movement (Fig. 8c,d). First, the locations of neurons activated before and/or after the initiation of motion in the left or right hindleg were either established from raw imaging data by visual judgment of the experimenter (Supplementary Movie 8) or were extracted with a cross-correlation method (Supplementary Fig. 10a). As a result, activity in the M2 area on the contralateral side was higher than that on the ipsilateral side (Supplementary Fig. 10a). Therefore, the right and left hindleg movement accompanied the premotor neuronal activity mainly on the contralateral side of the cortical area, which roughly matches the hindleg position in the secondary motor cortex as estimated previously^21^. However, both these detection methods have intrinsic problems, as visual judgment can easily miss important signals, and screening by cross-correlation can also miss the responses of neurons that do not appear in every trial, but may still be physiologically important. Thus, a more comprehensive quantitative analysis was sought by merging the image data of multiple events (11 left leg kicks, 10 right leg kicks). The maximum FI value during the period (3 sec) before or after the kick was identified for each pixel, and then this value for each pixel was subtracted by the maximum FI value of the same pixel during three 6-sec periods with no leg movement. The subtracted maximum FI value for each pixel was mapped in the right column images of Fig. 8e where the timing of the emergence of each maximum value was color-coded either in green or red depending on whether it appeared before or after the initiation of leg movement, respectively. We further took into account the frequency of these peaks appearing in all kick events, and Fig. 8f shows the time windows before or after the initiation of leg movement during which each peak appeared (see Supplementary Fig. 10b,c for the detailed process). Different levels of color intensity represent the frequency of appearance, and the different colors represent the timing of the maximum peaks. These results also support the conclusion that leg motion is preceded by premotor activity mainly in the contralateral M2 area as a starting point, and as shown here, the activity started within a period of between −2 sec and −1 sec. This timing supports results using the other methods described above, although the distribution does not strictly follow the conventional M1/M2 division, with the activity propagating from the anterior to the posterior poles and from contralateral to bilateral areas broadly. Further qualitative analysis also supported these results (Supplementary Fig. 10d–g), with a cell grouping method based on raw imaging data across the entire motor cortex finding that the cell assembly presenting the pre-motor activities is the main contributor of the lateralized activities both before and after a specific movement (middle graphs of Supplementary Fig. 10d,e). This result suggests that the lateralized activity of the premotor active cell assembly ensures the later lateralized motion. In conclusion, these results indicate that the HLC can visualize specific cell assemblies representing premotor activity in the whole motor cortex (Fig. 8g).

## Discussion

In this study, we describe a wearable imaging system with wide-field, deep-focus optical capabilities that will enable comprehensive physiological analyses of the broad and deep area making up the cerebral cortex.

We admit that raw imaging movie captured by the HLC contains multiple noises, since the HLC detects the signals from the cell bodies, dendrites and axons in the various layers due to its deep-focus optical system. However, the application of the NMF analysis (Fig. 5) or other methods as described in Fig. 8e,f and Supplementary Fig. 10 to the detected signals enabled grouping of the neighboring pixels with high activity correlations as putative cells and excluded the noise spots from such ROI detection as the cells.

Furthermore, comparison of the images by the HLC and 2-photon microscope from the GAD67-GCaMP6f mouse in which the fluorescent cells are more sparsely distributed than in the CaMKIIa-G-CaMP7 mouse showed that the levels of the signal information are similar between the images of the HLC or 2-photon microscopy in the awake mouse brain in terms of the number and distribution of fluorescent ROIs (cells) (Supplementary Fig. 8g). These data support that by application of proper image processing algorithm, we can resolve the information of activities of individual cells while keeping their relative positions among each other are kept.

In particular, long and continuous (in one day) or longitudinal (over multiple days) observation of the brain in the same individual under free-moving conditions can be performed easily with the HLC. In fact, we observed the light response of the neurons in the visual cortex over multiple days (day1 and 18, or day 11 and 20 after operation). These advantages make the HLC an easy-to-use tool for initial survey analyses prior to using more sophisticated systems for higher resolution studies. To enable better detectability and spatiotemporal resolution, we will need to further improve the optical system and image sensor. In addition, to compensate for the lack of depth information available with HLC imaging, transgenic lines expressing a fluorescent Ca^2+^ indicator could be used to image specific cortical layers or cell types.

Various applications for imaging in the present study revealed the visualization of orientation selectivity encoded in individual excitatory neurons within the whole visual cortex of one hemisphere, and also revealed specific cell assemblies representing activation of the entire bilateral motor cortex during volitional behavior output. The firm attachment of the HLC imaging system to the head of a freely moving mouse enables the stable visualization of the neuronal activities in the same field of view at high reproducibility over many days, and the lightweight property of entire imaging system allows the mouse to move freely with relatively a small amount of stress, also permits the simultaneous use of multiple HLCs (Supplementary Fig. 3, Supplementary Movie 10).

Various volumetric imaging technologies have been developed recently. For example, in multi-photon microscope technology capable of deep observation, a 2P-RAM (2-photon random access mesoscope)^22^ provides a wide field of view with high spatial resolution. However, it is impossible to eliminate the time lag for scanning multiple areas in the large field. A wearable endoscope equipped with a light-field optics^23^ provides 3D imaging with no time lag but with sacrifice of spatial resolution, but it requires a large amount of calculation for the reconstruction of 3D image. On the other hand, a method of 3D imaging by combining a light-field or HiLo (highly inclined and laminated optical sheet) microscopy with a stage system that keeps up with fast motion of an animal was developed^24,25^. Under the necessity to carry out high-speed tracking at high precision in a fixed direction and the limitation in the size of observation field of view and depth, these system have been applied for the whole-brain Ca^2+^ imaging of the freely moving animals with relatively small bodies such as zebrafish larvae. Some of the above methods require expensive and large-scale equipment.

In contrast, among the currently available various wearable optical imaging tools, the HLC is positioned as a unique wide-field, volumetric, and low-invasive imaging device for fluorescence imaging. The HLC in this paper provides a wide field of view and improved detection capability of deep signals. There is no time lag in imaging by the image sensor unlike the galvano scanning system. The laser of the excitation light source provides higher S/N with higher light density than the LED. The spacer apparatus with cranial window can be easily attached to the head with simple surgical operation and enables longitudinal observation over multiple days. Simple mechanisms of the HLC are suitable for commercial production.

Fluorescence change was maintained to be observable as long as 57 days after operation (Supplementary Fig. 8e,f). Judging from the observation by stereo microscopy in Supplementary Fig. 2b, meanwhile, the clarity of the cranial window was kept unchanged for 100 days in the same individual. Therefore, we assume that longitudinal Ca^2+^ imaging is possible for at least 3 months. Because we stopped imaging and monitoring within 3 months, it might still be possible to continue observation beyond this period.

Although only a few animal experiments were conducted to showcase the utility of the microscope in this paper, in the near future, longitudinal imaging using the wearable HLC tracking the same neurons at the same site under free-movement conditions will help to reveal neuronal activity that is specific to various behavioral tasks. Additionally, the simultaneous use of multiple HLCs will facilitate investigation of cognition and behavior through neuronal activity imaging of the global sensorimotor system in animals, especially by a combination of electrophysiology and optogenetical applications.

## Methods

### Development and construction of a wearable imaging system

The wearable HLC imaging system is composed of two major parts, the camera and the spacer (Fig. 1a). The two parts can be separated or combined by using the O-ring as a fastener hooked on their side protrusions. The cylindrical spacer body has a cranial imaging window (object plate) at its base that makes direct contact with the surface of the cortex. The spacer is attached to the mouse head, and the camera is combined with the spacer when imaging is performed. The detachable wearable camera helps with the long-term housing of experimental mice by keeping them free from movement restriction by an electrical wire (Supplementary Movie 1). The camera module (DCT, Co., Japan) has a CMOS (complementary metal-oxide semiconductor) image sensor chip (1/13″, 480 × 640 pixels, pixel size = 1.75 × 1.75 µm; OmniVision Technologies Inc., USA) and a deep-focus optical system. The imaging area is adjustable, and the bottom diameter of the typical cylindrical spacer body is 7.5 ± 0.5 mm. Although the design of spacer body is changeable, typically the height of the spacer body is 9.0 ± 0.5 mm (long spacer in Fig. 5), in this case, the volume of spacer part is approximately 500 mm^3^. The thickness of head cap and gasket is 1.0 mm ± 0.5 mm each, therefore, the total volume of the HLC is approximately 600 mm^3^. The HLC is lightweight (typically 0.9 g) and does not disturb the natural behavior of a small mouse such as individuals from the C57BL/6 line (Supplementary Fig. 3d,e).

The HLC has an LD for the excitation light and an absorption filter for the emission light. The camera was constructed by firmly attaching the micro camera and LD to the head cap with epoxy resin. For green and red fluorescence imaging, the high pass absorption filter set at >500 or >520 and >560 or >580 nm, respectively (Fujifilm, Co., Japan) was attached in front of the lens, and a blue or green LD (450–460, 488 or 530 nm; OSRAM, Co., Germany) was used, respectively. The LD along with the LSD (Optical Solutions, Co., Japan) and the copper heat sink is driven by the LD driver (Supplementary Fig. 5a,b). The glass object plate (0.525 mm thickness; Matsunami Glass Ind., Ltd., Japan) was firmly attached to the bottom of the spacer body. The head cap and the spacer body were made based on 3D-CAD data by cutting an acrylic plate with a lathe machine.

The image data were transferred to a computer via a USB cable (Supplementary Fig. 1a), displayed on a monitor, and saved as an AVI movie file using free software, AmCap (Microsoft, Co., USA). For Ca^2+^ imaging, the image sensor was typically driven at <30 fps to ensure sufficient sensitivity, stable data transfer and preservation, and real-time presentation on the monitor. The ΔF/F of the mechanical noise when measuring inorganic matter was below the detection limit of 8-bit data. ImageJ (supplied by the National Institutes of Health, USA) was used for all image data analysis. Graphic art works were performed using the free software, DesignSpark Mechanical (3D CAD; computer-aided design, Radiospares Components, Inc.) and GIMP (GNU Image manipulation program, Free Software Foundation, Inc.).

### Verifying the optical specifications of the HLC

The HLC has deep-focus optics and its working distance (WD) can vary by adjusting the lens barrel. The lens position change can also vary with the field of view size at the same time. When the lens barrel is twisted and pulled out for a short WD, the view field becomes small. In contrast, when the lens barrel is twisted and pressed for a long WD, the view field becomes large. If the position of the camera is adjusted properly such that the WD fits the object, the HLC can obtain either a narrow or wide field of view arbitrarily, as shown in Fig. 2a,b. The minimum field of vision is 2.00 × 2.67 mm at 2.70 mm of WD, and here the maximum resolution was 8.33 µm/lp (line pair), 4.17 µm/pixel. The maximum field of vision is 10.0 × 13.3 mm at 15.0 mm of WD, and here the minimum resolution was 41.7 µm/lp, 20.8 µm/pixel.

There is a physical limit to the barrel adjustment range, and if adjusted to an extreme WD value, light aberration cannot be corrected and the peripheral portion of the image is distorted in a pincushion pattern. For instance, with 6.65 × 8.87 mm imaging, the central part of the image can cover 6.65 mm vertically, whereas that of the periphery vertically covers 6.40 mm, which means DTV (television distortion) = (6.65–6.40)/6.40 × 100 = 3.91 [%]. Additionally, the central and peripheral parts of the image horizontally cover 8.86 and 8.67 mm, respectively. Therefore, the optical distortion is 2.78 %, calculated as follows: distortion [%] = [(actual half diagonal distance) − (predicted half diagonal distance)]/(predicted half diagonal distance) × 100 = √[(6.65/2)^2^ + (8.86/2)^2^] − √[(6.40/2)^2^ + (8.67/2)^2^]/√[(6.40/2)^2^ + (8.67/2)^2^] × 100. The pincushion distortion will be suitable for observing the convex cortex, but not the barrel distortion (see also right panel in Supplementary Fig. 7b).

Next, the depth of field (DOF) was calculated. DOF is defined by an associated resolution and contrast, and is estimated by a single value calculated from the diffraction limit as a theoretical approximation; however, it is difficult to make a genuine comparison because many imaging lenses are not diffraction limited. Therefore, the only way to truly determine DOF is to use a test target. Normally, even if a lens has infinite focus theoretically, the spatial resolution of the image sensor (density of the photo-diode pixel array) is limiting for DOF. Firstly, the line spread function (LSF) of the HLC in the case of 6.65 × 8.87 mm imaging (resolution; 27.7 µm/lp, 13.9 µm/pixel) was analyzed by using a line chart (Fig. 2c,d), and then based on the results of the LSF, the square wave response function (SWRF) was calculated as follows:

$${C}_{out}(u)=\frac{\frac{1}{2}({I}_{max}(u)-{I}_{min}(u))}{\frac{1}{2}({I}_{max}(u)+{I}_{min}(u))}=\frac{{I}_{max}(u)-{I}_{min}(u)}{{I}_{max}(u)+{I}_{min}(u)}$$

$$SWRF(u)=\frac{{C}_{out}(u)}{C(0)}=\frac{[\frac{{I}_{max}(u)-{I}_{min}(u)}{{I}_{max}(u)+{I}_{min}(u)}]}{[\frac{{I}_{max}(0)-{I}_{min}(0)}{{I}_{max}(0)+{I}_{min}(0)}]}$$

*C*_*out*_(*u*) is the output contrast of rectangular wave pattern at spatial frequency *u*. *I*_*max*_ and *I*_*min*_ are the values obtained by converting the intensity into the dose at each square wave of the LSF. As a result, the SWRF is 24.7 [lp] at a cut-off point of 0.03, as generally used by many optical manufacturers (Fig. 2e). That SWRF value corresponds to the position at 600 pixels in LSF (Fig. 2d). In the line chart, the line pair width is 0.912 mm. Therefore, DOF is 24.7 × 0.912 × √3/2 = 19.5 [mm].

Finally, to calculate an effective spatial frequency at the distorted edge of the imaging field, the SWRF (a rectangular wave response function) was corrected to the modulation transfer function (MTF; sine wave response function), calculated with the correction via Coltman’s formula^26^. MTF is a measure of an imaging lens’s ability to transfer contrast from the object plane to the image plane at a specific resolution, and is expressed with respect to image resolution (lp/mm) and contrast (%). Typically, as resolution increases, contrast decreases until a cut-off point, at which the image becomes irresolvable and grey. The formula is shown below.

$${MTF}(u)=\frac{\pi }{4}\sum _{k=1}^{\infty }{B}_{k}\frac{{\rm{SWRF}}\{(2k-1)u\}}{(2k-1)}$$

$${Resolution}=\frac{1}{lp\,/\,mm}\times 1000$$

If the total number of prime numbers in (2*k* − 1) is *m* and the number of types of prime numbers is *n*, *Bk* = 0 when *m* > *n*, and *Bk* = (−1)^*n*^(−1)^*k*−1^ when *m* = *n*. For the calculation of MTF, up to the fourth term of the expansion formula was used according to a previous verification^27^. As a result, MTF resulted in an effective spatial frequency of 15.5 lp/mm where MTF = 0.03 (Fig. 2f), producing an effective spatial resolution of 64.5 µm/lp. Therefore, the estimated minimum effective spatial resolution was 64.5 µm/lp at the distorted edge of the imaging field, which is 2.3 times that of 27.7 µm/lp at the center of the visual field in the case of 6.65 × 8.87 mm imaging (resolution; 27.7 µm/lp, 13.9 µm/pixel). According to the above results, although actual measurement values will fluctuate depending on the measuring environment and optical distortion, a discriminable minimum light spot is presumed as approximately two times blurred at the edge of the field of view due to natural optical aberration.

### Quantitative imaging analysis using fluorescent beads

After craniotomy, fluorescent beads (F21010, green fluorescent FluoSpheres, polystyrene; Thermo Fisher Scientific Inc., USA) were implanted into the cortex of the anesthetized mice at different depths by using a needle and micromanipulator (Fig. 3). The bead diameter of 15 µm was similar to the general cell size. The stereomicroscope has a general objective focus lens, whereas the HLC has a deep-focus lens. In Fig. 3, the field of view of the HLC is 3.80 × 5.06 mm, 1 pixel = 7.917 µm. A general objective lens can handle a bright image because the F value is smaller than the deep-focus lens, and a sharper image was obtained by focus imaging in Fig. 3b than in Fig. 3c; however, the DOF was shallow. In contrast, with the HLC, the fluorescence derived from every bead can be detected even at an 800 µm depth, suggesting that the detectability of fluorescence by the deep-focus lens is apparently higher than that for a conventional focus lens. In the left 2 images of Fig. 3d, the position of the upper beads at 400 µm depth does not match exactly between the conventional microscope and the HLC. This is likely due to differences in optical distortion. Regarding the small 10 images to the right column in Fig. 3d, although these images are shown brightly for the sake of convenience in aligning and distinguishing shapes, the measurement was actually performed based on the raw data, with the quantitative results shown as a graph in Fig. 3e–g. In the microscope image, the FI and shape of each bead at the same depth are almost the same (left graph in Fig. 3e), supporting the accuracy of the experimental system. Thus, the deeper the position of the beads, the lower the FI becomes in the image taken by the conventional stereomicroscope, until the FI is reduced almost to background levels at 800 µm. In contrast, the decrease in FI with depth is mild for the HLC imaging, and a stronger fluorescent signal than for the conventional microscope was detected even at a depth of 800 µm. Compared at the same depth, the bead’s FI level and shape captured by the HLC are slightly variable. This can be reasonably explained by distribution differences in the excitation light (see also the image in Fig. 2h) and optical distortion (as mentioned above in Methods section for Fig. 2a). In fact, from the center of the field of view to the periphery, the intensity of the excitation light decreases and the size increases. It is possible to correct the above-mentioned variability mathematically, if necessary, in actual functional brain cellular imaging. Concerning the result of Fig. 3f, the deeper the position of the bead, the larger it becomes relative to its actual size in the conventional microscope images. In contrast, image sizes with the HLC are generally constant relative to depth. Specifically, the enlargement ratio at 50% FI with the stereomicroscope image against the actual bead size is 4.10 (at 100 µm), 14.6 (at 400 µm), and 17.6 (at 800 µm), compared to 4.75 (at 100 µm), 5.80 (at 200 µm), and 6.86 (at 800 µm) for the HLC imaging. Thus, both the decline in signal detection ability and the expansion of the outline seem to be less when using the HLC due to the deep-focus optics.

### Ca^2+^ imaging in 3D culture cells

Cell cultures in 2D and 3D, gene transfection, and drug administration were performed according to previous reports^9,28^. Hela cells, derived from a human cervical cancer, were transfected with the G-CaMP6 gene^12^ under 2D conditions. After transfection, cells were embedded in the collagen gel as an extracellular matrix to imitate brain structure as a brain phantom. Hela cells are activated by histamine through the histamine H_1_-receptor by producing intracellular Ca^2+^ increases^29^. Thus, when a histamine solution (final 5 µM) was applied to the dish, rising Ca^2+^ increases were detected by the HLC at 30 fps imaging (Supplementary Fig. 7a, Supplementary Movie 4). In contrast, the increase was abolished by 5 mM EGTA (Fig. 4b).

### Animal studies

All experiments and procedures involving animals conformed to the animal care and experimentation guidelines of the RIKEN Animal Experiments Committee (the approval No. H29-2-220) and Genetic Recombinant Experiment Safety Committee (the approval No. 2017-026, 2018-030). C57BL/6J (SLC Co., Japan), CaMK2a-tTA (Jackson 3010)^30^, TRE-G-CaMP7-2A-DsRed2^31^, and Thy1-G-CaMP7-2A-DsRed2 (Thy1-G-CaMP7)^32,33^ mice, aged 6-12 months, were used for the *in vivo* experiments (see details of surgical operation in Supplementary Fig. 2).

### Cell detection and signal extraction

In Fig. 6c, imaging data analysis was performed using custom codes in LabVIEW (National Instruments) and Matlab (Mathworks). Brain imaging movies were processed in the following steps to detect cell bodies and extract their temporal signals. First, for each pixel of the image, a variance value was calculated using the time signal of that pixel. Thus a variance map was obtained for each movie. This variance map has peaks and valleys of various heights, corresponding to the location cell bodies. This variance map was then cut horizontally at different heights to obtain 100–200 slices, depending on the data. Each of these slices showed presence of segmented ROIs. Cells with weaker activation were visible in slices cut in higher depth from the top, while highly activated cells were found in slices in lower depths. All ROIs with area in the range of 30–150 squire microns were gathered to form a preliminary set of cell-like ROIs. For each ROI, a 2-pixel-wide Gaussian filter was used to smooth the spatial distribution of the ROI. However, as in many cases same cells were detected multiple times in varying depths, temporal correlations among these ROIs were checked. ROIs having temporal correlation value >0.9 and some degree of spatial correlation were considered as same cells, and they were added to form one single ROI. By performing this task recursively, we obtained a second set of ROIs representing the cell bodies in the movie.

In the next step, we calculated temporal signals of these ROIs from the movie by taking weighted average of temporal signal of all the pixels in a specific ROI. As the intensity of the movie pixels varied due to position of pixels, ROIs has various levels of baseline intensity signals. Furthermore, in case of weakly activated cell bodies, actual calcium transient signal was not clearly visible due to temporally fluctuating baseline. Therefore, to eliminate baseline effect, we used a NMF (Non-negative Matrix Factorization) algorithm^14^ to extract actual activation of the cells while separating the spatio-temporal baseline of the image simultaneously by solving the following problem:

$$Minimize:{\Vert (F-W\times H-{S}_{b}\times {A}_{b})\Vert }_{2}$$

*F*: original image

*W*: spatial components corresponding to cells

*H*: temporal components corresponding to cells

*S*_*b*_: Spatial baseline

*A*_*b*_: Temporal baseline

With the application of the above mentioned algorithm, baseline removal and separation of temporal signals corrupted by spatial overlap of cells was achieved.

### Perceptual and behavioral tests

In Fig. 7, awake mice received either light stimulation or an 8-direction drifting grating with blue light stimulation using a light emitting diode (LED, peak wave length 470 nm; Stanley Electric Co., Ltd., Japan) driven by a function generator, or video stimulation using a computer monitor. C57BL/6 mice have high photosensitivity against blue light judging from their pupillary light reflex^34,35^. Thus, to increase the stability of repeated visual inputs to the mouse, the head of the mouse was fixed with a stereotactic instrument while its body was allowed to move freely (upper left image in Fig. 7a), so that the left eye was given the visual stimuli while the right eye was masked. All experiments were done in a dark room, and the imaging was started after 15-minute habituation. In Fig. 7a, the upper middle schematic diagram for the rodent brain was drawn with partial modification by reconstructing the transverse sections of the brain atlas^36,37^ (Supplementary Fig. 9a).

A single flashlight stimulation (rectangular pulse 0.1 sec) or 10-times repeated flashlight stimulation (rectangular pulse trains of 0.5 sec, 20 Hz, duration 25 ms, interval 25 ms) were applied to the mouse. Similarly, the 8-direction drifting grating (each drifting duration 3 sec, interval 6 sec, >0.5 cycle/degree^38^) was presented to the mouse. Ca^2+^ imaging was then performed using the HLC on the CaMK2a-tTA × TRE-G-CaMP7-2A-DsRed2 mice (CaMK2a-G-CaMP7) over the right visual cortical area. ROIs of 4 pixels were selected by visual judgment, and changes in FI were measured from the raw imaging data. Figure 7b represents the results of 12 ROIs of 2 mice in 6 trials, while Fig. 7c shows the results of 7 ROIs of 2 mice in 3 trials. All ROI positions are shown in the lower column images of Fig. 7a. In Fig. 7d, the left and right graphs plot the raw values obtained from 15 ROIs of 2 mice in 3 trials and 12 ROIs of 2 mice in 1 trial, respectively. ROIs of one mouse (ID: #2) among 2 mice is exemplified in Fig. 7e. In Fig. 7f, Data of 4 opposite angles ([0 and 180°], [45 and 225°], [90 and 270°], and [135 and 315°]) during the 3-second stimulation were maximized, and data of right angles were subtracted as below, ΔF [0 and 180°] = FI ([0 and 180°] − [90 and 270°]), ΔF [45 and 225°] = FI ([45 and 225°] − [135 and 315°]), ΔF [90 and 270°] = FI ([90 and 270°] − [0 and 180°]), ΔF [135 and 315°] = FI ([135 and 315°] − [45 and 225°]). Finally, these four different ΔF’s were assigned with different 4 colors (Green, Blue, Magenta, Yellow), and each ROI was given a pseudo-color by weighed superimposition of these 4 colors to represent the orientation preference of each ROI (Fig. 7f). Among these ROIs, those with top 3.5% of pseudo-color intensity are presented in Fig. 7g.

Regarding deconvolution (upper right image in Fig. 7a), diffraction point spread functions and iterative deconvolutions were calculated according to Dougherty’s algorithm^39^. In the present study, FI measurements were always extracted from raw data, and not from the deconvoluted data.

For the maximum points extraction (Supplementary Fig. 4a,b), light points were selected and counted from the image after subtraction of background with the parameter of a noise tolerance of 10 according to the ImageJ algorithm constructed by Michael Schmid (NIH, USA).

For the “restriction motion experiment” represented in Fig. 8, an apparatus was made to analyze neuronal activity in the mice derived from specific voluntary movement initiation. A cylindrical restraint tube was filled with urethane foam resin to fit the body shape of the mouse, and its external wall was painted with black ink. Hence, the body of the mouse is held firmly when it is inserted in the tube, and other parts of the body except the legs do not move as much, thus mildly restricting the mouse motion and sensation. Before beginning the measurements, the mouse was placed in the cylindrical restraint and their legs protruding from the tube were attached to splints, which functioned as body-worn foot levers. The splints could move according to the leg motions or could be made immovable individually by the fixing of bolts. The motion of the splint was followed by the movement of a line drawn outside the splint and captured by a web camera at 30 fps from both lateral sides. Then, Ca^2+^ imaging was performed after 15-minute habituation in 4 mice (ID: #3–6) using an HLC attached to the spacer protruding from the upper part of the restraint tube, at 20 fps (Fig. 8) or 10 fps (Supplementary Fig. 10a, Supplementary Movie 9).

A series of analyses comprising a “comprehensive quantitative analysis” (Fig. 8e,f, Supplementary Fig. 10b,c) and an “overall qualitative analysis” (Supplementary Fig. 10d–g) were also conducted as described in the respective figure legends. The maximization of FI in the imaging data, described as a maximum intensity projection in Supplementary Fig. 8, removed the basal random noise similarly to an averaging process, but without losing the information of relatively rare but important events.

## Kobayashi et al. Supplementary information

Kobayashi et al. Supplementary information

Supplementary Movie 01 (behavior).mp4

Supplementary Movie 02 (occipital cortex).mp4

Supplementary Movie 03 (Maximization).mp4

Supplementary Movie 04 (Hela cells).mp4

Supplementary Movie 05 (2-photon).mp4

Supplementary Movie 06 (Thy1).mp4

Supplementary Movie 07 (magnified).mp4

Supplementary Movie 08 (oscillation).mp4

Supplementary Movie 09 (premotor activity).mp4

Supplementary Movie 10 (freely moving, dual).mp4

## Data Availability

All relevant data are available from the authors.
