# Supplementary material for: Wide and Deep Imaging of Neuronal Activities by a Wearable NeuroImager Reveals Premotor Activity in the Whole Motor Cortex
Source: Sci Rep. 2019 Jun 10;9:8366. doi: 10.1038/s41598-019-44146-x (PMC6557893; doi:10.1038/s41598-019-44146-x)
Supplement: Supplementary file 1 — Kobayashi et al. Supplementary information [file 41598_2019_44146_MOESM1_ESM.docx]

**Supplementary information**

**Wide and Deep Imaging of Neuronal Activities by a Wearable NeuroImager Reveals Premotor Activity in the Whole Motor Cortex**

Takuma Kobayashi^1^*, Tanvir Islam^1^, Masaaki Sato^2,3,4^, Masamichi Ohkura^2,3^, Junichi Nakai^2,3^, Yasunori Hayashi^3,5,6^, Hitoshi Okamoto^1^*

1. Laboratory for Neural Circuit Dynamics of Decision Making, RIKEN Center for Brain Science, Wako, Saitama 351-0198, Japan

2. Graduate School of Science and Engineering, Saitama University, Saitama, 338-8570, Japan

3. Brain and Body System Science Institute, Saitama University, Saitama, 338-8570, Japan

4. Laboratory for Mental Biology, RIKEN Center for Brain Science, Wako, Saitama 351-0198, Japan

5. RIKEN Center for Brain Science, Wako, Saitama 351-0198, Japan

6. Department of Pharmacology, Kyoto University Graduate School of Medicine, Kyoto 606-8501, Japan

*Corresponding author. Tel.: +81 48 467 9713; Fax.: +81 48 467 9714.

E-mail address: [takuma.kobayashi@riken.jp](mailto:takuma.kobayashi@riken.jp); [dr.takuma.kobayashi@gmail.com](mailto:dr.takuma.kobayashi@gmail.com) (T. Kobayashi), [hitoshi.okamoto@riken.jp](mailto:hitoshi.okamoto@riken.jp) (H. Okamoto)

**
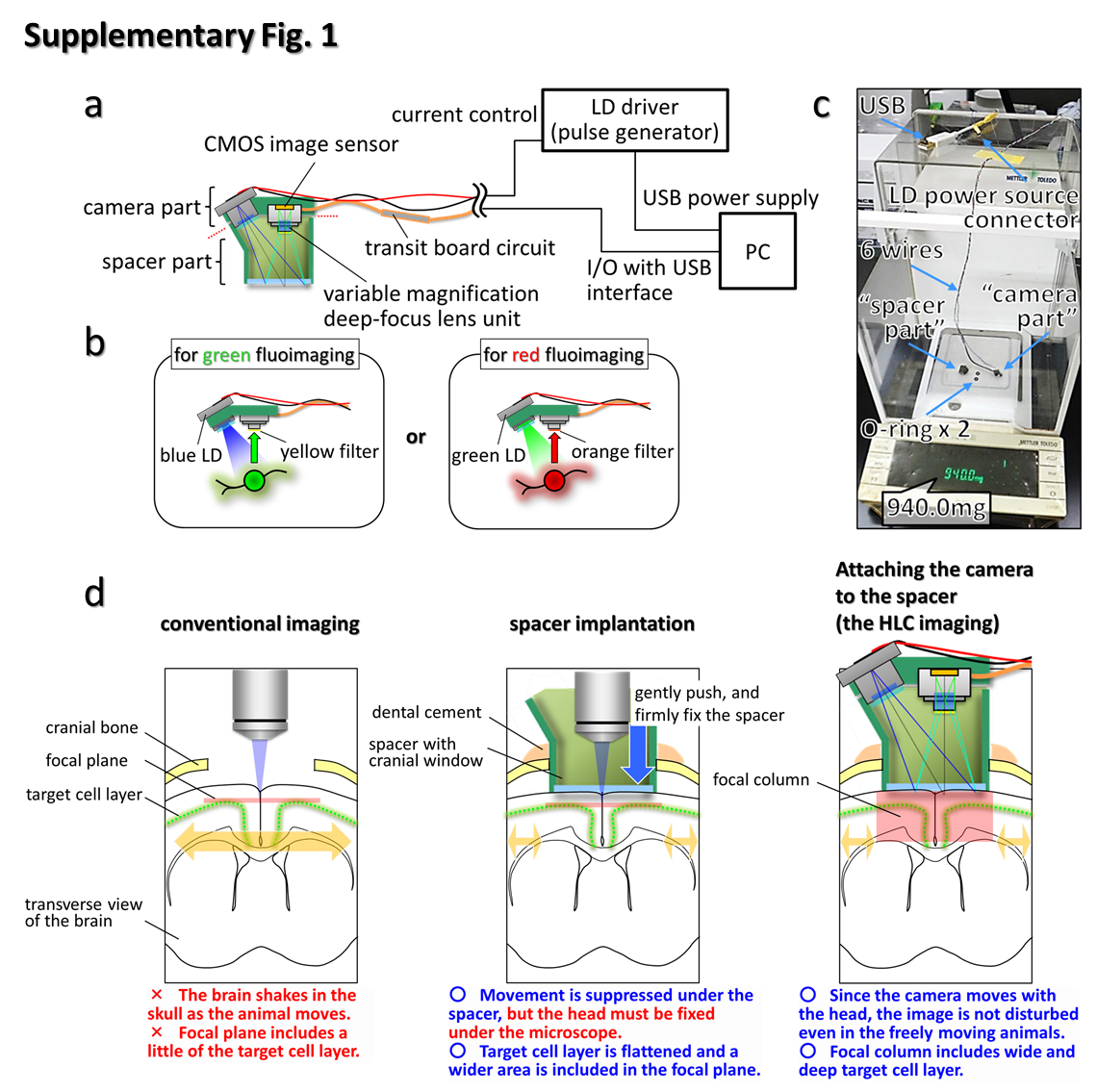
**

**Supplementary Fig. 1** The schematic diagrams of the HLC (head-mounted laser camera) imaging system.

**(a)** The schematic diagrams, including the block diagram and simplified ray diagram, of the main components of the HLC imaging system are shown. The digital imaging signal transfers through the transit board circuit, and inputs the PC (personal computer) via the USB interface. Inversely the CMOS image sensor is controlled by the output signals from the PC through the same line. The LD (laser diode) is driven by the LD driver (see for detail Supplementary Fig. 5a) whose current power is supplied by the PC via the USB interface. All components of the camera part and the spacer part are described in detail in Fig.1a. One camera part has one CMOS image sensor and one LD, and one spacer part has one cranial window. One camera part and one spacer part constitute one HLC imaging system. For the fluorescence imaging, the excitation light is irradiated from the LD via a LSD (light shaping diffuser), the emission light from the target object under the spacer part is captured by the CMOS image sensor through the absorption filter and the lens unit as a fluorescent light signal.

**(b)** The HLC imaging system can be applied to the fluorescence imaging for the different wavelengths by changing the type of the excitation light source and the absorption filter. For example, the blue LD and the yellow filter are used for the green fluorescence imaging, and the green LD and the orange filter are used for the red fluorescence imaging. Each actual HLC version is presented in the right pictures of Fig.1a.

**(c)** The weight measurement of all the constituent parts of a typical HLC is shown. As a result, the load of the mouse during the imaging is 0.94 g per one set of the HLC imaging system.

**(d)** The differences of in vivo imaging method using the HLC system are described. The left diagram shows the conventional imaging under the microscopy after the craniotomy. The brain can be regarded as a floating object in the cerebrospinal fluid inside the skull. Therefore, when the mouse moves, the brain shakes in the skull easily and the image is disturbed (orange bidirectional arrows in the schematic image means the brain shake). Additionally, the general objective lens has a narrow visual field and a thin focal plane in order to correct the optical aberration. Therefore, a small number of the specific neuronal cells in the specific layer that is bent along the curvature of the cerebral cortex of the mouse can be only observed in one focal plane. To relieve such difficulties of *in vivo* imaging, the use of the spacer apparatus will help. The middle diagram shows the imaging under the microscope with the spacer implantation. After the craniotomy, the spacer apparatus with the cranial window is implanted into the skull. The spacer is gently pushed to the cortex, and then firmly fixed with the skull by using the dental cement. As a result, the target cortical layer just under the spacer becomes flat and is held by the spacer. Therefore, more target neurons will be included in one focal plane, and the shaking accompanying the movement of the mouse will be suppressed. However, unless the head is still tightly fixed, the image by the objective lens under the microscope is disturbed by the animal movement. The HLC imaging system solves these problems and offers *in vivo* imaging under the freely moving condition. The right diagram shows the HLC imaging system. The camera part is attached on the spacer part which is implanted into the skull. Since the camera moves with the spacer and the mouse head, the disturbance of the captured image will be kept to the minimum even in the freely moving animals. Especially, a deep-focus lens that is equipped to the camera provides a “focal column” with the stacked focal planes, which will allow capturing many objects widely and deeply at once, unlike the conventional confocal imaging systems that takes time to acquire images requiring the xyz-axis scanning. As described above, due to the functional characteristics of the components, with some operational contrivances, the HLC imaging system is expected to contribute to imaging in awake animal under the freely moving condition and also to visualize the neuronal activity that cannot be observed under the anesthesia.


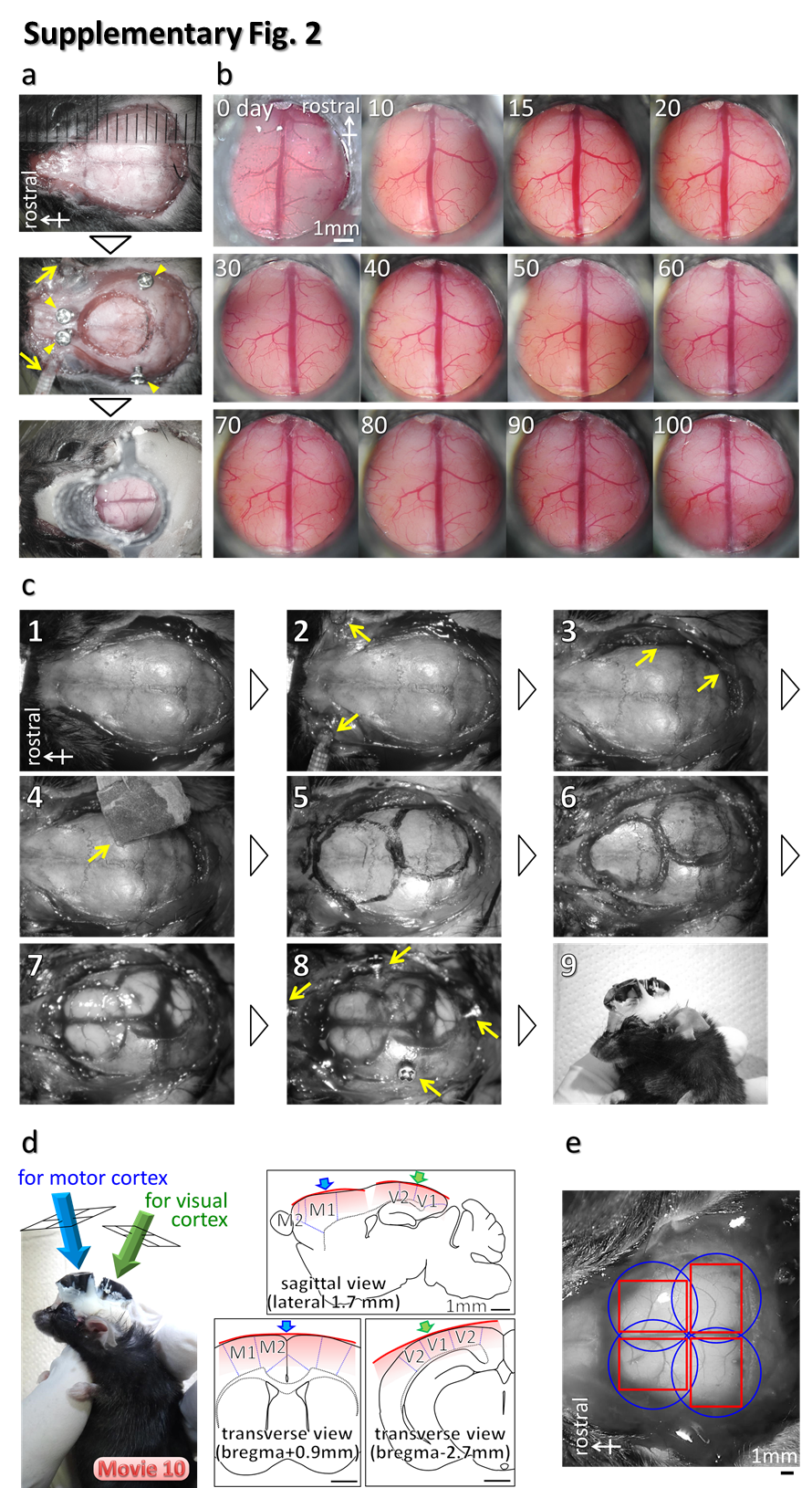


**Supplementary Fig. 2** The HLC allows long-term observation of the same brain area in the same mouse.

**(a)** Typical surgical operation and the process of the applying a single use of the HLC. The mouse was anesthetized with 2,2,2-tribromoethanol (125-250 mg/kg body weight^s1^) or a combination anesthetic consisting of 0.3 mg/kg medetomidine, 4.0 mg/kg midazolam and 5.0 mg/kg butorphanol^s2^, and was mounted on a stereotaxic instrument (Narishige Co., Japan). The head skin was scalped (upper panel). After craniotomy using a dental drill, the screws were inserted at bone positions around the hole to anchor the dental cement (yellow arrowheads in the middle panel). Eyelids were clamped with small forceps for protection (yellow arrows). The dura mater of the mouse was removed by using a hand-made sharpened tungsten needle gently and carefully. And then, the cylindrical spacer with a cranial window for imaging was attached to the mouse head by using dental cement (UNIFAST, GC, Co, Japan) (lower panel).

**(b)** Images show the temporal serial observation of the same mouse using a stereo microscope. Some part of the brain surface causes bleeding just after removing the dura although the extent of bleeding depends on operational skill. Bleeding was promptly stopped and the microvessels recovered in 4-5 days. Thereafter, the observation surface was kept clean for more than 100 days. During this long-term housing, neuronal activity could be visualized using the HLC and conventional 2-photon microscopy through the cranial window of the spacer apparatus (Supplementary Fig. 8).

**(c)** For multi-point imaging using two HLCs, surgical operation and the processes are shown. The detailed order of each process is described below;

1) Scalp removal.

2) Eyelid clamping using a small forceps for protection (yellow arrows).

3) Peeling part of the muscles of temporal and occipital regions (yellow arrows).

4) Removing the periosteum with water-resistant sandpaper (yellow arrow) (*e.g.* #600 waterproof paper file).

5) Marking the craniotomy position.

6) Drilling the skull by using a dental drill.

7) Removing the skull (optional: removing the dura mater).

8) Anchoring the screws (yellow arrows) (*e.g.* #0 pan head, M1.0 x 2.0 mm).

9) Attaching the cranial window parts with dental cement. Two cylindrical spacers with cranial windows were put on the cortex by using a precision manipulator to firmly support their positions, and then the spacers were fixed to the skull with dental cement. Besides, for behavioral testing under bright environments, black liquid rubber may be applied around the spacer and the dental cement for light interception.

**(d)** Example of installation of duplicate spacers for visual-motor imaging in the freely moving CaMK2a-G-CaMP7 mouse (Supplementary Movie 10). Blue or green arrows indicate the direction of observation from the HLC for imaging including the whole motor area of bilateral cortex or the whole hemispheric visual area, respectively (left picture). The right panels show the directions of observation and the thick and convex area of imaging by the HLC (red line and graded red area, see also right image in Supplementary Fig. 7b) both in the sagittal (upper panel) and the cross (lower panels) sections. Use of multiple cameras allows the broad area imaging of the curved cortex at cellular resolution (>4.17 µm/pixel).

**(e)** Schematic image of the application of four HLCs. Blue circle and red square indicate the position of the spacer and the imaging area, respectively. The quadra imaging achieves simultaneous imaging of the entire cerebral cortex. As mentioned in Fig. 2a, one HLC can capture a wide field of view of 10.0 x 13.3 mm. This size is almost equal to one brain of the mouse. However, in that case, the resolution is lowered to 20.8 µm/pixel, and the sight from a single direction distorts the image of the spherical brain near its periphery. With multi-point thick imaging, it is possible to image at a high resolution with low distortion along the curvature of the cortex.

**
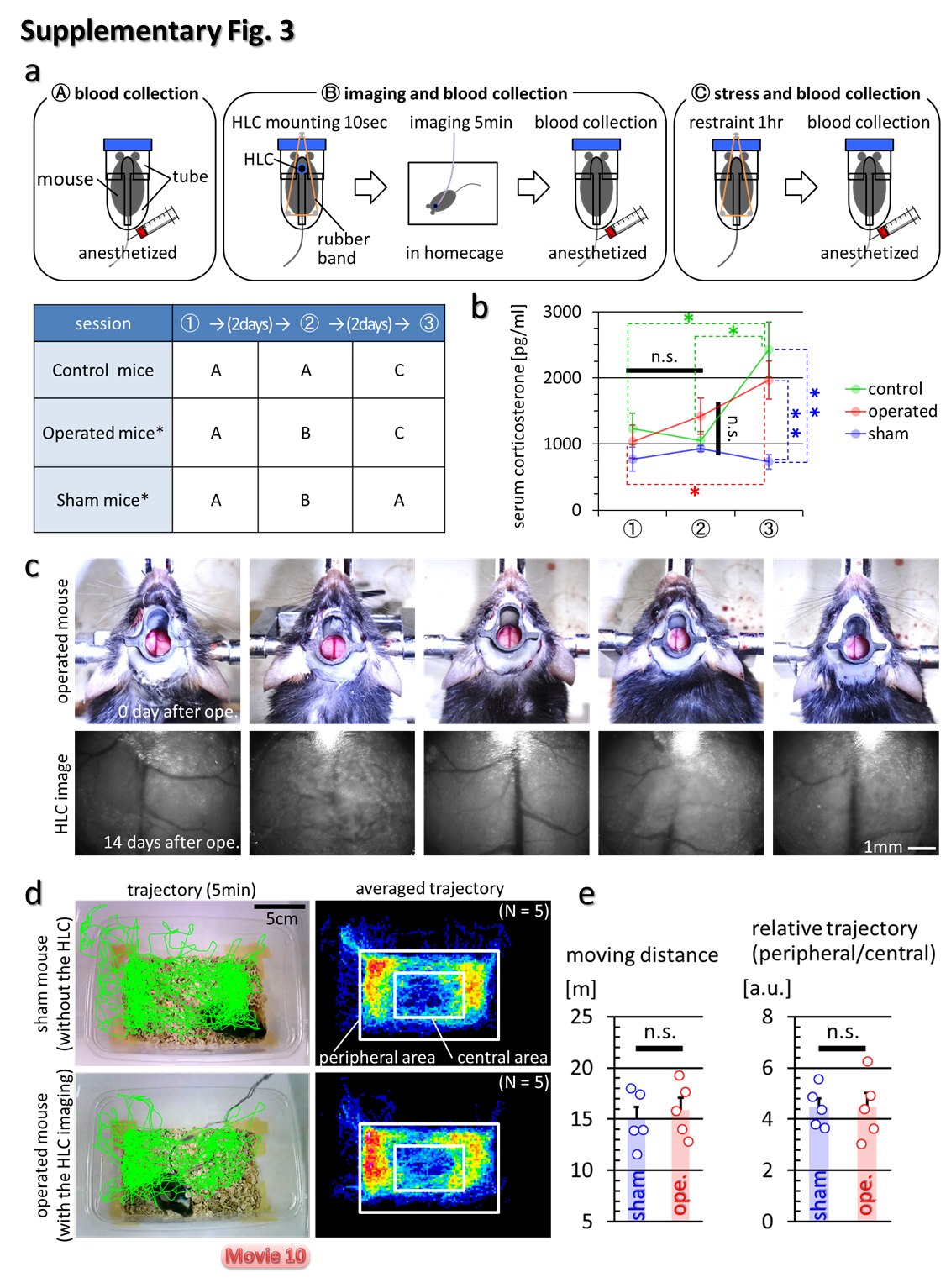
**

**Supplementary Fig. 3** The imaging of neuronal activities by the HLC attached to the head of freely moving mice causes no significant increase of stress, and does not affect the locomotor activity and the behavioral pattern.

Blood-based and behavioral assessments of stress caused by wearing the HLC were performed.

**(a)** Corticosterone produced in response to stress was measured. Schematic images of experimental procedures are shown in A-C (upper images). These 3 different experiments were conducted in 3 sessions every 3 days for 3 groups of mice (each N = 5, total 15 mice) (lower table). 8-10 months old C57BL/6JJmsSlc male mice were purchased (Japan SLC, Inc., Japan). A group of mice with an asterisk were given the 5 minutes daily handling for 1 week prior to the experimental session. “Operated mice” were attached with the spacer apparatus with cranial window on his frontal cortical area by surgical operation performed 2 weeks before this experiment. “Sham mice” are the wild-type mice which underwent the same treatment as in the procedure B but were not mounted with the HLC.

**(b)** The result of ELISA assay of the serum corticosterone level is shown. All procedures were performed according to the manufacturer’s protocol (Corticosterone ELISA Kit, Cayman Chemical Company, USA). Error bar is a standard error of the mean. T-test was performed after F-test for judging significance. Single asterisks mean there is the significance of p < 0.05 between session 1 and 3 (p = 0.0271), session 2 and 3 (p = 0.0137) in control mice, and between session 1 and 3 (p = 0.0213) in operated mice. Double asterisks mean there are significance of p < 0.01 between control and sham mice (p = 0.0067), operated and sham mice (p = 0.0049) in session 3. There is no significance (n.s.) between session 1 and 2 in all mice group, and between control, operated and sham mice in session 2. These results indicate that the HLC mounting and imaging process causes no significant increase in stress in the mice.

**(c)** These photos show the individual operated mice and their frontal cortical image taken by the HLC under the freely moving condition (“ope.” means the surgical operation).

**(d)** The locomotor activity in the homecage from which the lid was removed was examined. Left column images indicate the examples of the moving trajectory for 5 minutes by a sham and an operated mouse (Supplementary Movie 10). Right column images indicate an averaged trajectory of 5 sham and operated mice. The averaged trajectories were binned with 5 x 5 pixels, and shown with pseudo-color. The bottom of the homecage was subdivided into the central and peripheral areas as indicated in the figures.

**(e)** The numerical analyses of (d) are shown in the graphs. Error bar is a standard error of the mean. Each circle indicates the value of each individual. There is no significance (n.s.) between the sham and operated mice in the moving distance or the distribution of the trajectory. These results suggest that HLC mounting and imaging process does not affect the locomotor activity and the behavioral pattern.


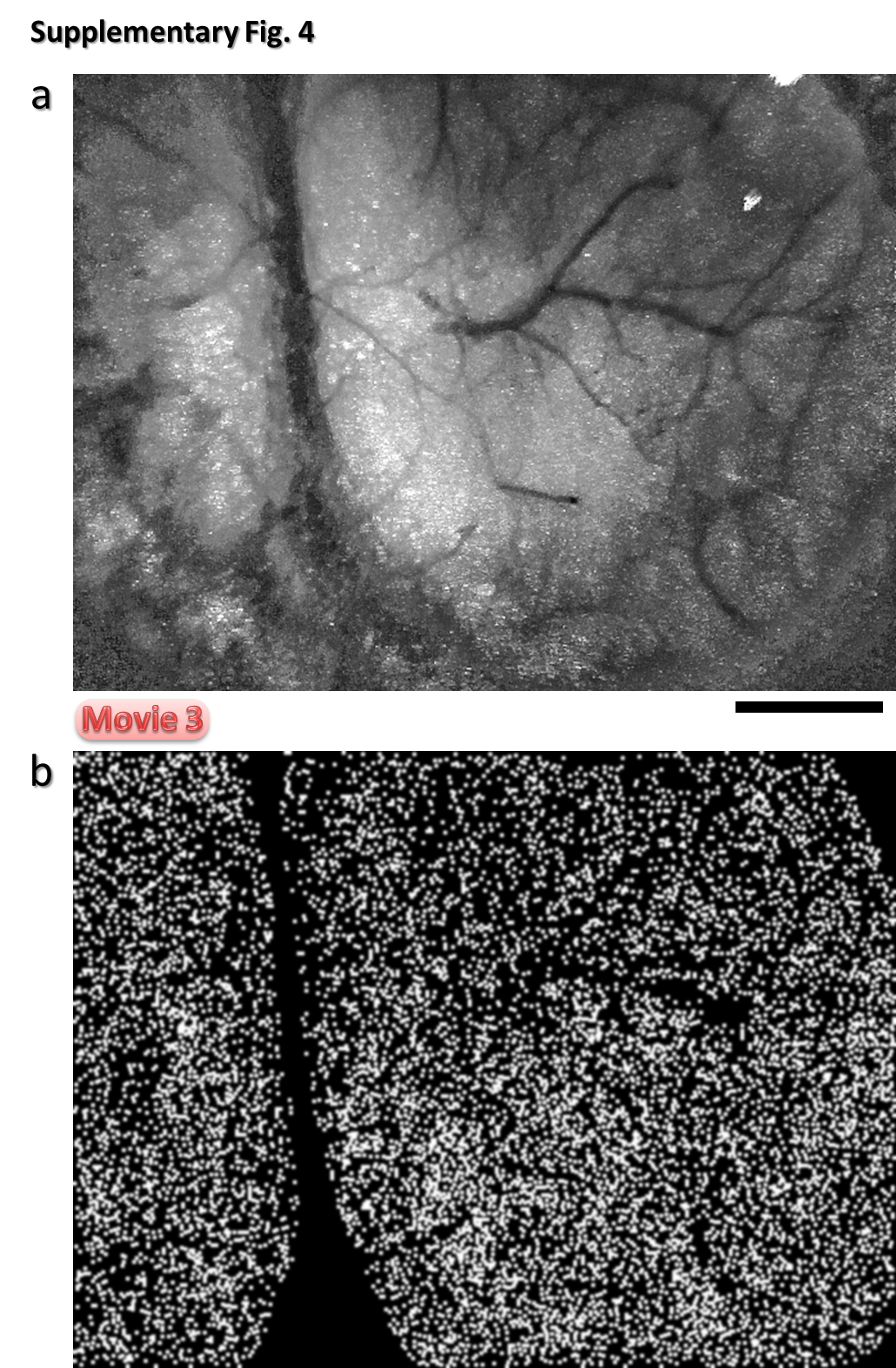


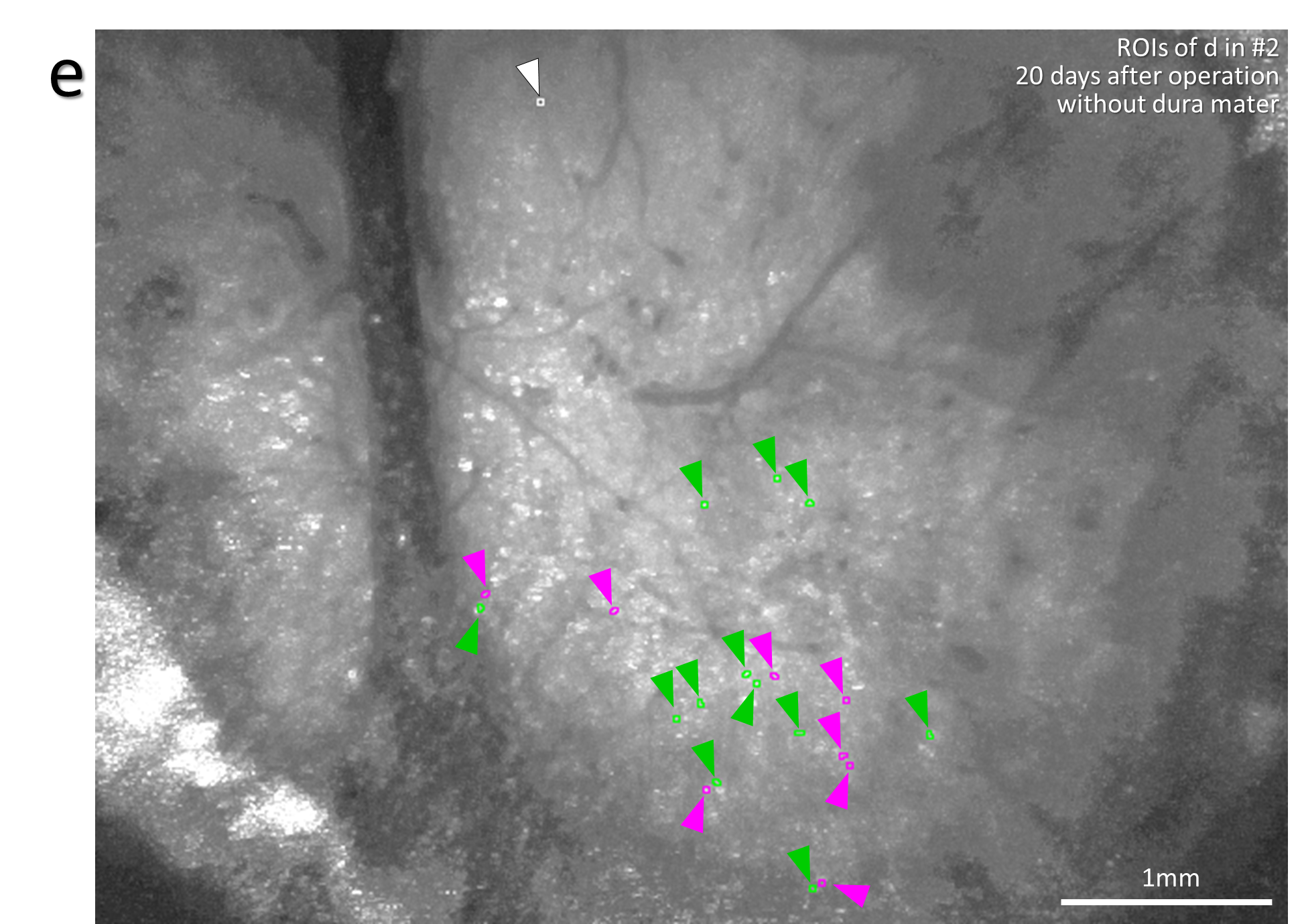

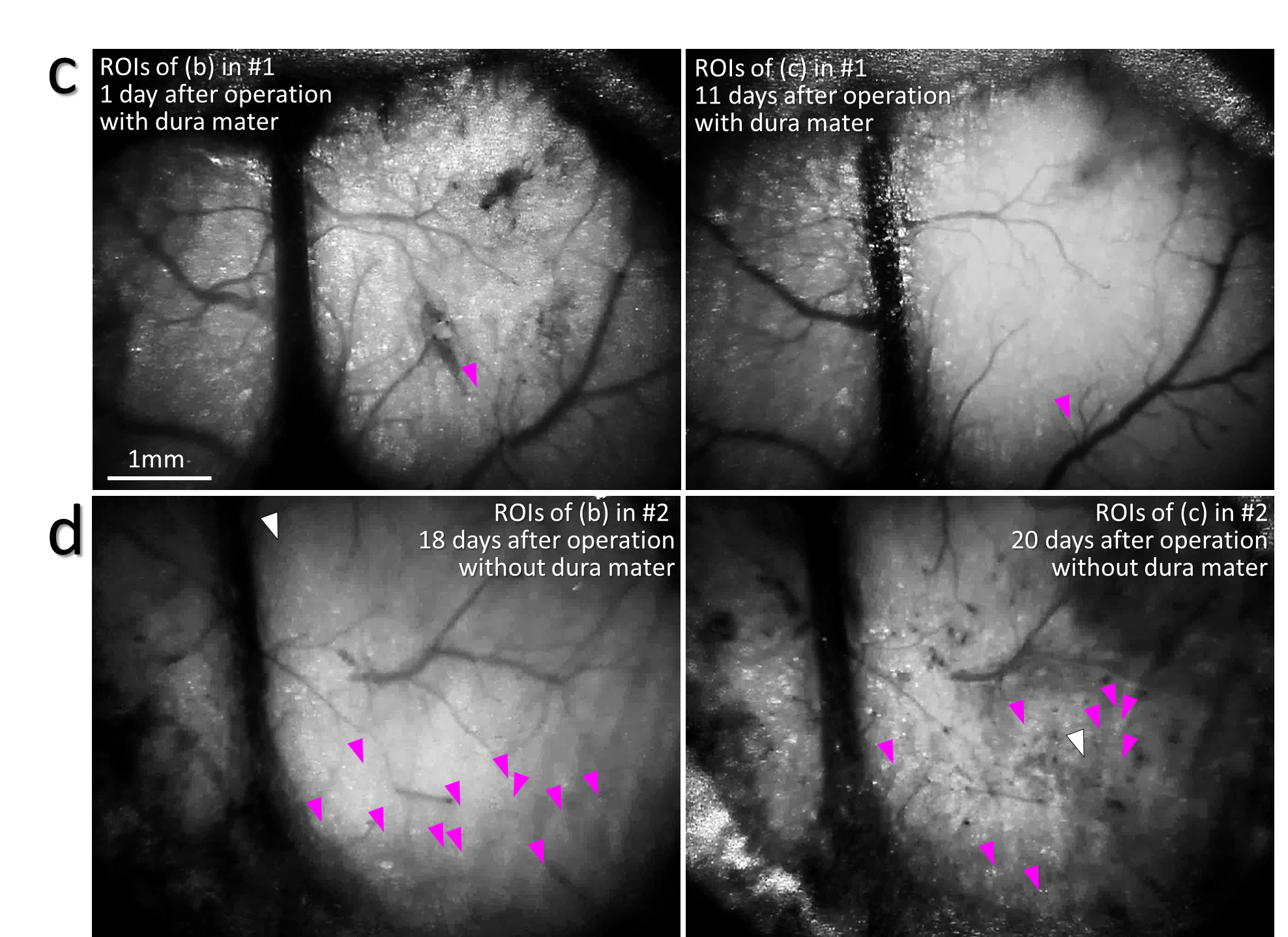


**
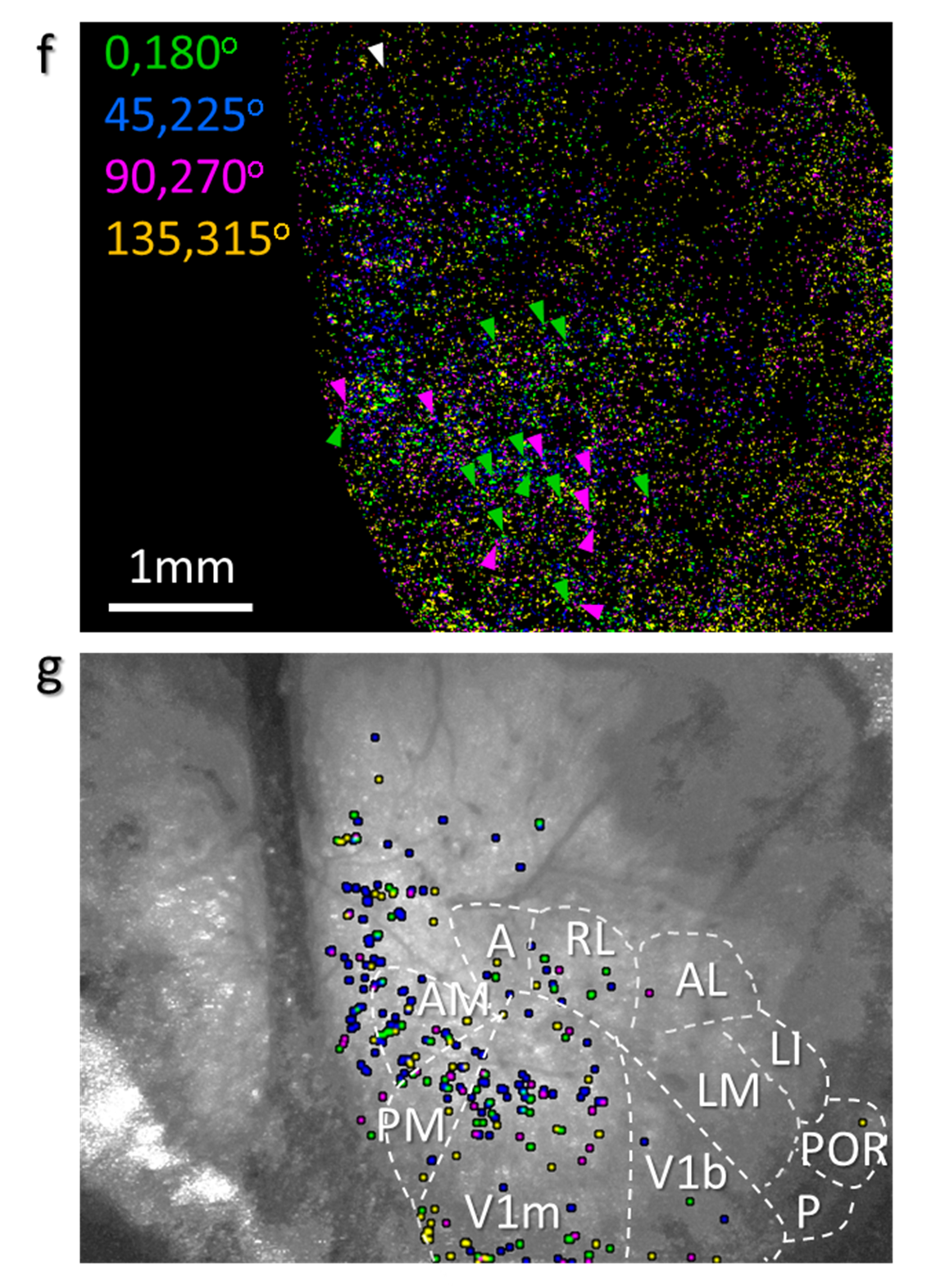
**

**Supplementary Fig. 4** Automated selection and counting of discriminable blinking light spots.

**(a, b)** Ca^2+^ imaging was performed by the HLC on the visual cortex of the CaMK2a-G-CaMP7 transgenic mouse (Fig. 7). In order to estimate how many excitatory neurons can be detected and discriminated in the occipital area including the visual cortex by the HLC, the merged image was analyzed by counting the number of peak fluorescence spots (see for detail Methods section). 8 raw movies for 1 min were taken by the HLC and merged by maximization. A representative 10 seconds of the merged movie is shown in Supplementary Movie 3, and the results from where all frames were merged by maximization is shown in (a). Bar indicates 1 mm. The maximum points of the fluorescence spots of (b) are shown in (a). 9352 light spots could be discriminated within a single field of view by one HLC (4.25 x 5.66 mm, 30.7 x 10^4^ pixels, 8.8 µm/pixel, 1-6 pixel/spot in (a), 5 pixel/dot in (b)).

**(c, d)** are magnified images of the lower panels of Fig. 7a.

**(e-g)** are magnified images of Fig. 7e-g.


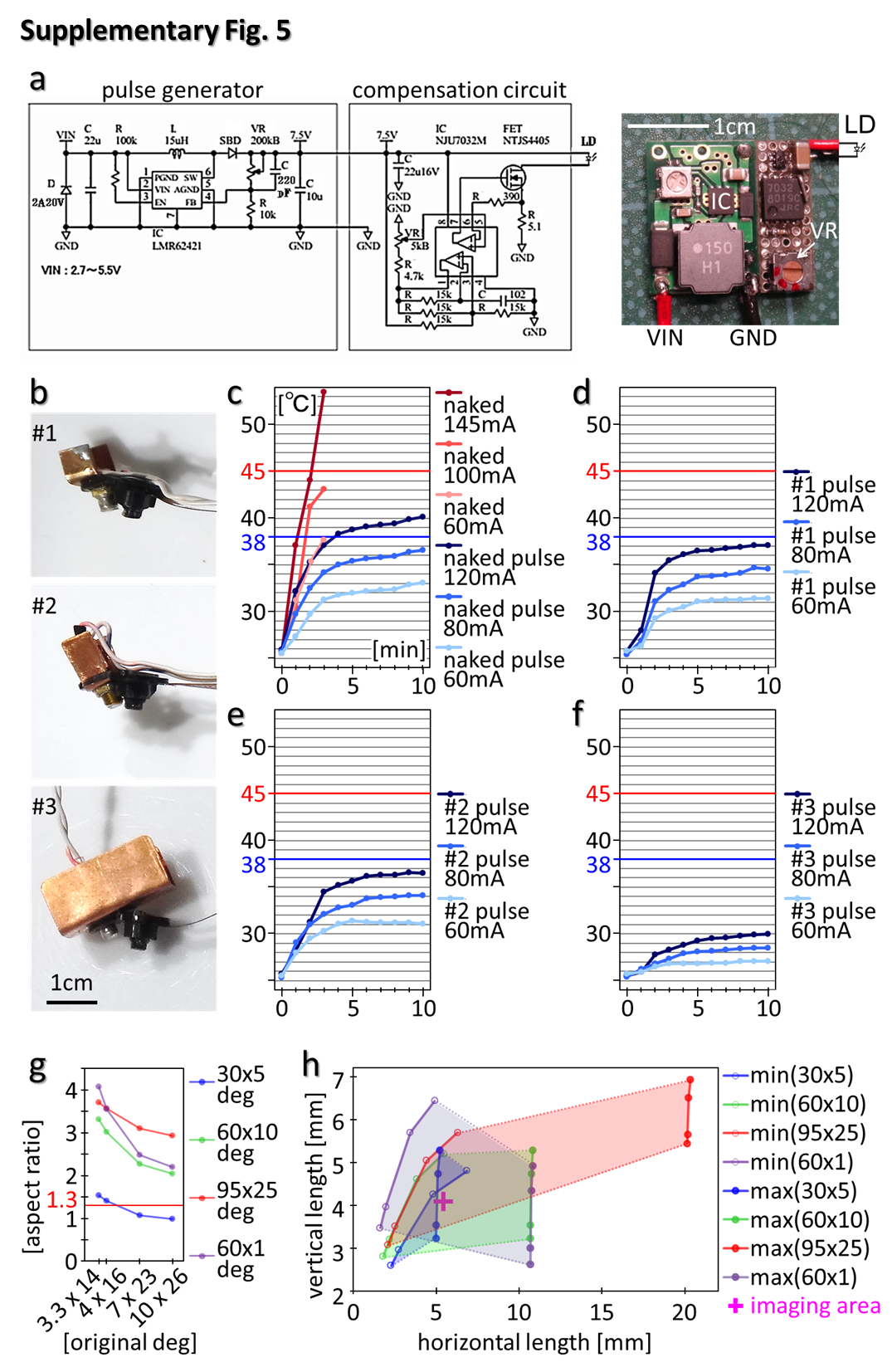


**Supplementary** **Fig. 5** Components: a pulse generator to drive a laser diode (LD), an external heat sink structure to suppress the temperature rise of LD, and a light shaping diffuser (LSD).

**(a)** The LD driver and its circuit diagram. To prevent a temperature rise of the LD due to continuous illumination, a LD driver (ImageTech, Co., Japan) was regulated by a pulse generator which turns the LD on and off at a high-speed. In the circuit diagram, the left box shows an oscillator and the right box shows a compensation circuit. 32 kHz pulse is stably supplied to the LD. Since a source voltage allows 2.7-5.5 V input, the LD driver can be driven by an external power supply unit or also directly connected to the USB power supplying wire, which contributes to the miniaturization and simplification of the entire system. A CMOS operational amplifier was used in the compensation circuit for a constant current. MOS-FET (metal-oxide-semiconductor field-effect transistor) was used for an output enhancement as a switching element. Output power for LD can be controlled by a variable resister (potentiometer) (VR, white arrow in right image). Therefore, it is possible to irradiate excitation light with a preferable output according to the observation target during the imaging. VIN, input voltage; GND, ground; C, capacitor; R, resistor; L, inductor; D, diode.

**(b)** A heat sink of variable size was attached to the LD of the HLC. #1/ #2/ #3 shows 146.7/ 194.7/ 889.2 mm^2^ heat sinks, respectively.

**(c)** The temperature change of the LD (PL450B, OSRAM, Inc., Germany, threshold current 30 mA, maximum optical output power 100 mW, threshold current 30 mA, operating current <145 mA) was examined. The temperature of the metal housing of the LD attached to the heat sinks was measured using a thermocoupled probe. The room temperature was 25.5 ^o^C. Blue or red horizontal lines in each graph indicates 38 or 45 ^o^C respectively, as a typical mouse’s body temperature or as a presumptive limit temperature at which irreversible damage is caused to the cell. When a constant current of 60, 100, or 145 mA was applied to the “naked” LD (no heat sink), its temperature rapidly exceeded 38 ^o^C (c). In contrast, when a pulsed constant current (32 kHz) of 60, 80, 120 mA was applied using a pulse generator, the temperature of the naked LD increased more slowly, finally reaching a plateau level after 10 minutes, thus showing the effectiveness of the pulse drive for stable lighting by the LD and the prevention of a rapid temperature rise.

**(d-f)** The temperature of the LD with the heat sink was measured. These results indicate that the cooling efficiency by the heat sink is higher as its surface area increases. The temperature of the LD with every type of heat sink reached a plateau without exceeding 38 ^o^C, even if they were driven at 120 mA. Therefore, the heat sink structure is effective for cooling the LD. Also, the time of LD with the #1 heat sink temperature to reach 95 % of its maximum temperature was more than twice as fast as that of the naked LD. From a practical viewpoint, we decided to use the #1 sink in our *in vivo* experiments.

It is necessary to change the current value of laser diode (LD) according to the observation target. The experimenter needs to dim the LD while observing the fluorescence of the target during the experiment. As far as using the HLC (image sensor is OV7690) with the CaMK2a-G-CaMP7 mouse, it was mainly used at under 60-80 mA in most cases. At this situation, the temperature is 33.0 - 36.5 ^o^C and it is below the body temperature of the mouse, so we do not need the heat sink (c) although type #1 was mainly used for *in vivo* imaging in this paper. Since the LD is not in contact with the mouse and it is installed away from the mouse, even if it reaches 40 ^o^C at 120 mA, it is considered that there is practically no problem if lighting is limited to short time. On the other hand, it is considered preferable to use a heat sink if it is expected to be used for longer period at approximately 90 mA or more. In this condition, LD is expected to exceed the mouse body temperature (depending on the room temperature).

The heat sink suppresses the temperature rise above the room temperature, and shortens the time to reach the plateau, so it stabilizes the laser light quickly. Therefore, the experimenter can shorten the standby time associated with turning on and off the LD. In summary, if the experimenter emphasizes weight reduction of the HLC, it is not necessary to attach the heat sink. While, if the experimenter wishes to give priority to shortening the standby time, it is effective to attach the heat sink.

**(g, h)** The LD is used as the excitation light source of the HLC. The laser light from LD has a flat shape (lower leftmost panel in Fig. 2h). Therefore, the irradiation beam was processed by using a light shaping diffuser (LSD). In the right panel of Fig. 2g, the LD was tilted 30 degrees from the light axis of the camera and the paper was illuminated from a distance of 8 mm, which is the same condition as actually used in the HLC. As a control, we used a white LED that projected a round light bundle. The projected images by the white LED with a circle lens or LD (PLT5 488, OSRAM, Inc., Germany) were taken in the dark by a digital camera (COOLPIX P7100, Nikkon, Inc., Japan) with ISO100, exposure time 1 second, and fixed focus. The oscillation threshold of the LD, PLT5 488, is 30 mA, and the maximum limit current is 150 mA. According to the specification, the original beam divergence changes in the range of 4 x 16 - 7 x 23 (at 75 mA) - 10 x 26 degrees (deg) depending on the amount of applied current. Since the LD is usually driven at 40-80 mA for *in vivo* imaging, the distribution of LD at 40 mA was first analyzed. In the upper line of Fig. 2h, the projected circle light by the white LED was processed to a horizontal ellipse shape with various LSDs; original, 30 x 5 deg, 60 x 10 deg, 95 x 25 deg, 60 x 1 deg. Similarly, the figures in the lower line show that the projected vertical elliptical light from LD was processed to horizontal ellipse shape. In the result of the light projection using the 60 x 10 deg LSD, the distribution of a yellow-colored area with a half-value intensity was within an inner red square which corresponds to the typical view field of the HLC (4.0 x 5.3 mm). Therefore, we thought that the light distribution processed by the 60 x 10 deg LSD most closely matches the region of the imaging field of the HLC.

Continuously, the aspect ratios of the diffusion angle of the projected beam by the various LSDs were calculated depending on the original divergences of the LD at different currents (g). And finally, the various projected ranges by the various diffusion angles were simulated (h).

The graph in (g) indicates the aspect ratio of the vertical and horizontal projected angle in each diffusion angle LSD (indicated by different colored lines) plotted against various divergences of the original LD beam at different currents. X-axis values are plotted according to the horizontal degree of the original LD beam. The aspect ratio of the imaging area of the CMOS, *i.e.* 640 x 480 pixels, is 1.33 (led line).

When the LSD is applied, the diffusion angle is approximated by the following formula: √[(light source divergence angle)^2^ + (LSD diffusion angle)^2^], and the diffusion range is approximated by the following formula : (the distance to irradiated surface) x 2 tanθ, where θ is the half of the diffusion angle. In this experiment, the light source position was tilted by 30 deg horizontally. Therefore, the distance to the irradiated surface was 8 mm x 2/√(3), and the horizontal diffusion range was also corrected by multiplying 2/√(3). From the measurement result based on the projected original image in Fig. 2h, the original beam divergence of LD was 3.3 x 14 deg at 40 mA. In the case of 3.3 x 14 deg, the shape tendency calculated by the corrected angle at various aspect ratios of the LSD coincides with the actual distribution in Fig. 2h.

As a result, a 30 x 5 deg LSD is the most preferable and 60 x 10 deg LSD is the secondarily preferable for all current ranges of the LD.

The graph in (h) indicates the various projected ranges (vertical, horizontal length) by the various diffusion angles of the LSD (indicated by different colored lines) at the minimum or maximum current of LD. The diffusion angle increases or decreases according to an increase or decrease of the current value. The minimum projected ranges with various LSDs by the minimum current were calculated based on the yellow-colored area of projected image of Fig. 2h, and are shown as “min (LSD variation)” on the right side of the graph. Similarly, the maximum projected ranges were estimated based on 3.3 x 14, 4 x 16, 7 x 23, 10 x 26 deg as a maximum original divergence, and are shown as “max (LSD variation)”. The four dots in the data for each LSD show the results in order of the current value. The colored area for each type of LSD indicates the projected range that can change from the min value to the max value. “Imaging area” (magenta cross in the graph) indicates the typical HLC imaging area (4.0 x 5.3 mm). As a result, all tested LSDs except the 30 x 5 deg LSD can cover the imaging area.

From these results, the 60 x 10 deg LSD is the most preferable for the LD of the HLC under the various assumed usage conditions.


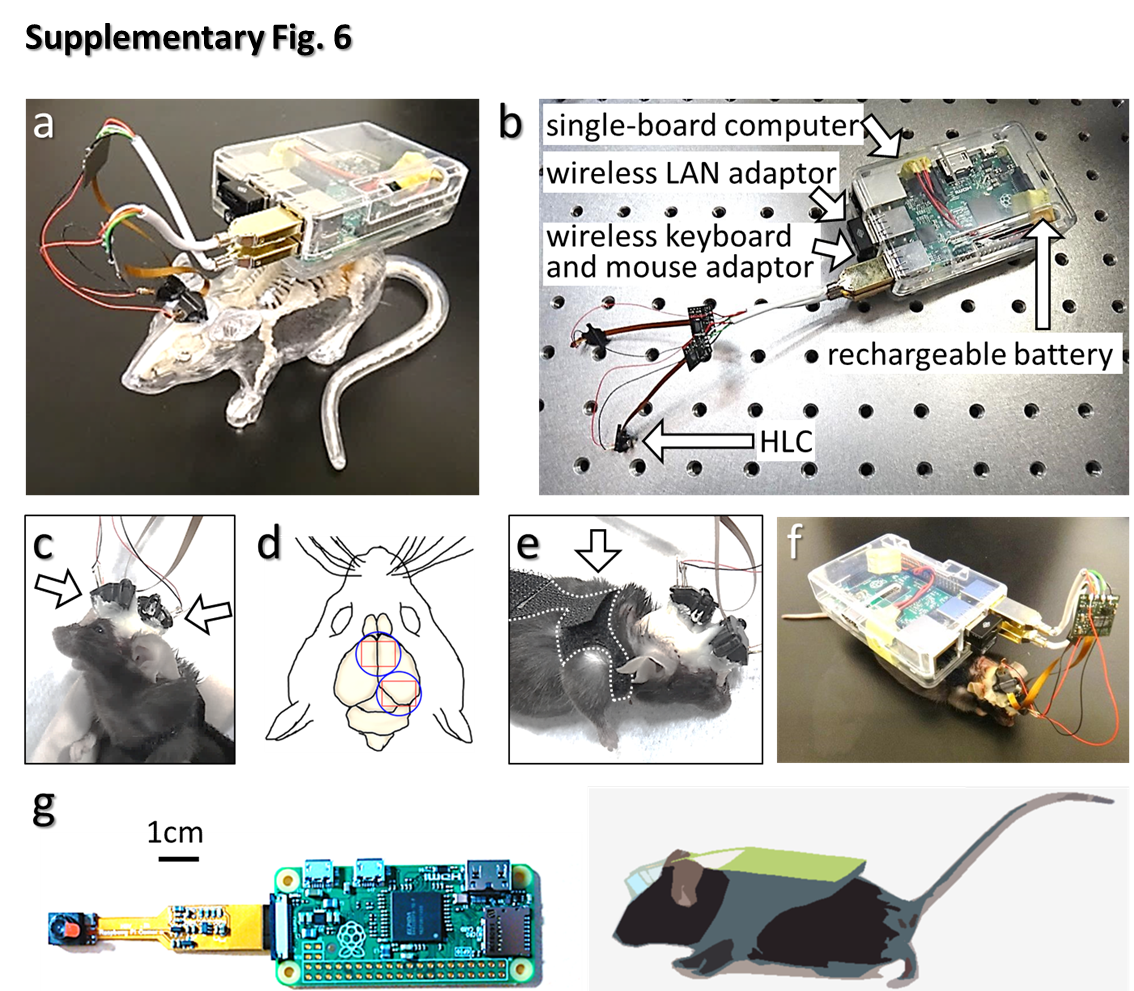


**Supplementary Fig.** **6** Wearable on-demand wireless imaging system using single-board computers.

An on-demand device is preferable for real-time observation of natural behaviors. Therefore, we constructed several types of wearable wireless units by using single-board computers. The CMOS image sensor used in the HLC is more compact and can work on lower power than a CCD (charge-coupled device), and is suitable for wireless control.

**(a, b)** Examples of a wearable on-demand wireless imaging system are shown. Dual HLCs were applied to the visual cortex of both hemicephalons on the rodent maquette (a). A Raspberry Pi 2 model B (Raspberry Pi Foundation, UK) was used as a main single-board computer (b). This multi imaging system is composed of the HLCs and adaptors for wireless LAN, keyboard, and mouse. Therefore, it can autonomously perform imaging and recording, and accept control from other PCs as a slave and send real-time video by remote operation. The wireless multi imaging system whose weight is 48 g can run for approximately 6–10 minutes with a rechargeable battery. The operating time can be extended depending on battery capacity.

**(c)** An example of multiple uses of the HLC is shown (see for detail Supplementary Fig. 2c, d).

**(d)** A schematic image of the applied position of (c) where the frontal or occipital cortical area including the motor or visual cortex is shown. The blue circle and red square indicate the spacer position and the imaging area, respectively.

**(e)** The image indicates the mouse wearing a vest to attach the device. In order for the mouse to move freely, it is necessary to carry the unit on its back (see also schematic image in (g)).

**(f)** An example of the wireless multiple imaging system (a) applied to a C57BL/6 line mouse. However, the weight and size of this type of system are slightly large and heavy for the tiny body of mouse. For instance, the ICR mice line that has a relatively large body size, a rat, or other large animals will be suitable for this wireless system.

**(g)** The system can be scaled down by using Raspberry Pi Zero as shown. Raspberry Pi Zero is smaller and lighter than Pi 2, approximately 1/12 by volume. Therefore, this type of wearable wireless imaging system will be more suitable for small animals such as the C57BL/6 line mouse.


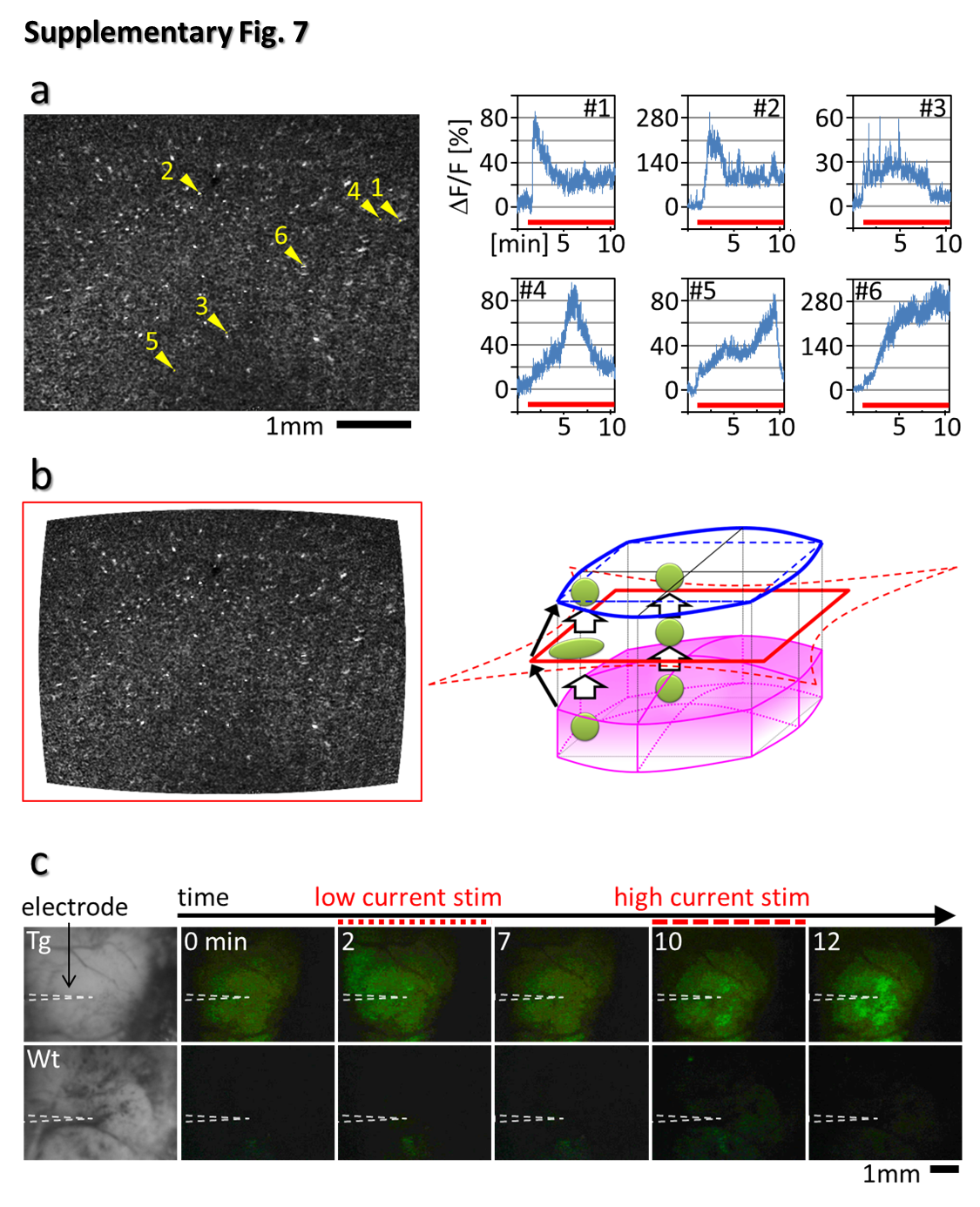


**Supplementary Fig. 7** The HLC can visualize intracellular Ca^2+^ dynamics of individual cells in 3D culture and artificially evoked neuronal activities by electrostimulation in the deep cerebral layer.

**(a)** The results of individual analysis of the Ca^2+^ imaging by the HLC of the 3D cultured Hela cells that were transfected with the G-CaMP6 gene (Fig. 4a) are shown. The 106 fluorescence spots in the imaging movie (10 frame/sec, 10 min) were selected randomly as 1 or 4 pixels ROI. The mean of fluorescence intensity (FI) changes at each ROI and the change rate of FI (ΔF/F) was calculated as (F-F_0_)/F, where F_0_ is the mean FI of the first 10 frames in each ROI. The maximum ΔF/F was 693.3 % in these ROIs. The positions of a representative 6 ROIs are shown by the arrowheads with numbers in the image of (a), and their ΔF/F are shown in the graphs and movie (Supplementary Movie 4). The fluorescence intensity was increased after histamine administration (red line in the graph). The graphs are arranged in chronological order of the timing of the ΔF/F peak appearance. The results indicate that the fluorescence functional cell imaging that represent the Ca^2+^ dynamics within each cell can be performed by the HLC at single-cell resolution.

For traditional 2D culture conditions without embedding into the extracellular matrix gel, the FI of the Ca^2+^ indicators usually promptly starts to increase just after histamine administration, or within 2 minutes at the latest (supplemental information in the previous study^9^). Under the 3D culture condition, however, the response may be delayed for longer periods depending on individual cells, sometimes as long as more than 10 minutes. Such a delayed response might depend on the depth of each cell because there should be a natural time lag in the diffusion of the histamine into the interior of the gel.

**(b)** The left image of (a) was corrected with DTV (television distortion) -3.9 % according to the result of Fig. 2a. Red square line indicates the image silhouette of (a). The right schematic diagram shows the correction process of the image. Magenta block indicates the thick convex image area of the HLC. The magenta gradient schematically represents the difference in fluorescence detection ability according to the result of Fig. 3. A red line indicates the image silhouette of (a) that is taken by the HLC. The red broken line indicates the positive pincushion distorted actual image of the original object (magenta block). The blue line indicates the negative barrel compensated image of the red line, and also indicates the restored original image silhouette of the object (magenta block). The blue broken line indicates the predicted non-compensated image of the blue line, in other words, the positive barrel distorted image of the blue line. The distortions of the schematic images are enhanced for the ease of visual understanding. Black arrows show the same corner of each image. Green circles indicate the cells. The distortion is smaller near the center of the original object, while the distortion is larger near the edge of the original object. Therefore, the cell shape that was enlarged at the periphery in the image taken by the HLC is corrected to the original size in the restored image (left picture).

**(c)** Ca^2+^ imaging was performed by the HLC on the somatosensory cortex of the Thy1-G-CaMP7 transgenic mouse (Fig. 4c, d). We performed Ca^2+^ imaging with the HLC for evoked activity before Ca^2+^ imaging of other physiological neuronal activities. In this experiment, an HLC equipped with 450-460 nm LD, 30 x 5 deg LSD and >520 nm emission filter was used. After craniotomy, a coaxial electrode (TOG207-078, Unique Medical Co., Ltd., Japan) was inserted into the somatosensory cortical layer 5/6 at around 500-600 µm depth using a micromanipulator. Then, a low or high tetanic stimulation (3 or 10 mA biphasic square waves, 100 Hz, duration 150 µs, interval 10 ms, 3 sec.) was applied by using a stimulator (Isolated pulse stimulator 2100, A-M Systems Inc., USA). The time lapse imaging data of the *in vivo* experiment (Fig. 4c, d) on the Thy1-G-CaMP7 mouse (Tg) or wild type mouse (wt) for the negative control are shown. The electrode was inserted into the cortex from the side after the craniotomy. The HLC was attached on the cortex and then the Ca^2+^ imaging was performed. When the stimulation was applied, the FI increased around the electrode in the Tg mouse, and the response was greater after high current than low current stimulation. In contrast, similar evoked signals were not detected in a wt mouse. These results indicate that the HLC can detect evoked neuronal activities in the cortex. Especially, it is thought that these changes of the FI mainly reflect the activities of the layer 5/6 pyramidal neurons and its neurites, because the Thy1 promoter is activated in those neurons predominantly.

In the wild-type case, slow and slight increases of the FI were also detected after stimulation. It is thought that this reaction reflects the intrinsic flavoprotein fluorescence in mitochondria^s3^. The excitation peak is near 450 nm, therefore, the endogenous flavoprotein imaging depending on neural activity is also accessed by the HLC although the amount of FI change is much smaller, and the response speed is much slower than G-CaMP. Such flavin imaging would allow the functional neuronal imaging in the completely intact brain.


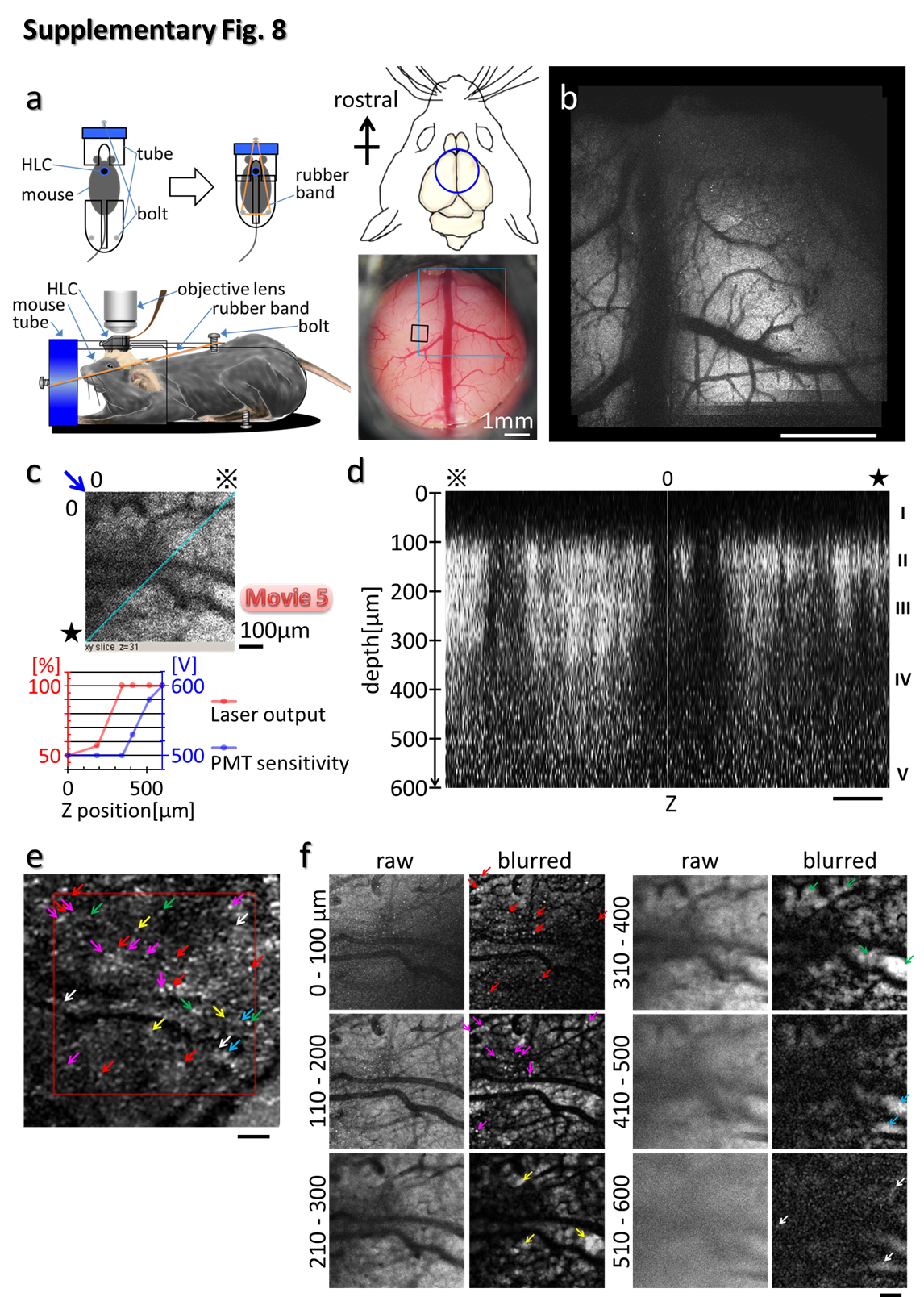


**
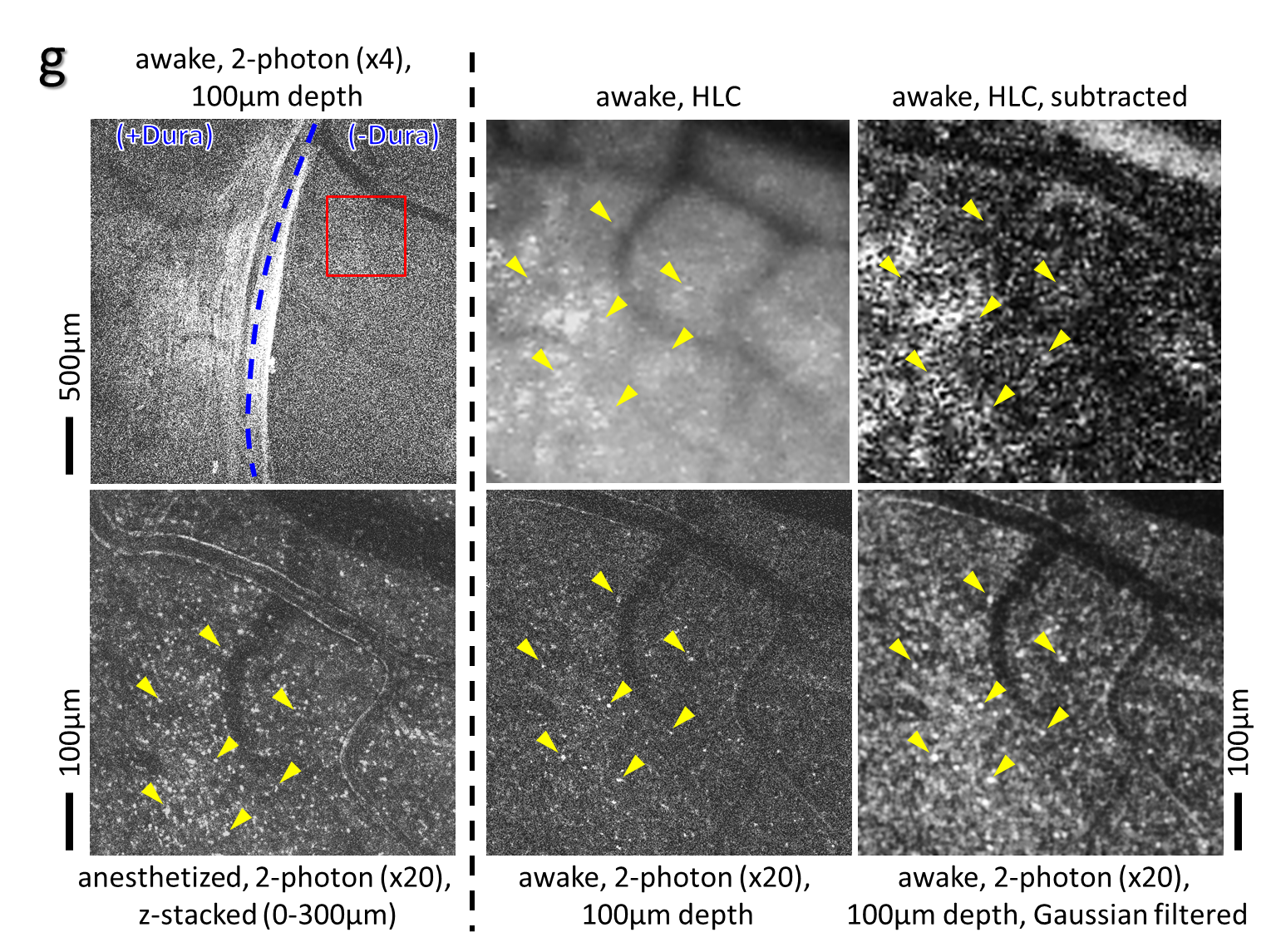
**

**Supplementary Fig. 8** The spacer apparatus with the cranial window allows the HLC and 2-photon microscope to perform chronic Ca^2+^ imaging at the same position in the same mouse.

**(a)** Fixation of the mouse head with the HLC on the head. After craniotomy and removal of the dura mater, the CaMK2a-G-CaMP7 mouse (mouse ID: # 3) was installed with the spacer apparatus with a cranial window on the frontal cortical region (see for detail Supplementary Fig. 2a, b). Then, fluorescence imaging was performed by using a 2-photon microscope (FVMPE-RS, Olympus, Co., Japan) at 5-6 days (b) and 33 days (c, d, f) after the surgical operation. The mouse was held in a plastic tube with the head fixed by the attachment of the HLC spacer to the hole of the tube (schematic image of left panel). The schematic image of the right upper panel indicates the location of the cranial window (blue circle). The right lower image was taken by a stereo microscope through the cranial window. Blue or black square indicates the imaging area of (b) or (c, d, f), respectively.

**(b)** The fluorescence imaging was performed in the awake condition with a 2-photon microscope using a galvano scanner. The view field was 3.18 x 3.18 mm, 512 x 512 pixels, 6.21 µm/pixel, and images were captured 10 times at 1.01 sec/stack at the depth of 300 µm. The acquired imaging data were merged. Misalignment caused by mouse movements between stacks was corrected manually, and a “maximum intensity projection”, *i.e.* a creation of the image where each pixel contains the maximum value over all images in the stacks, was performed. The result is shown in (b). Each fluorescent spot should represent excitatory neurons. Bar indicates 1 mm.

**(c)** The fluorescence imaging was performed with a 2-photon microscope using the galvano scanner in the anesthetized condition (upper panel). The view field is 636 x 636 µm, 512 x 512 pixels, and the images at 0.917 sec/stack were captured to a depth of 600 µm every 10 µm. The image shows a horizontal section of the cerebral cortex at the 31th stack, that is, a depth of 300 µm. The blue line and blue arrow indicate the position of the vertical section of the cortex and the direction of view in (d). The reference marks of two type asterisks correspond to the positions shown in (d). The lower graph indicates the imaging setting. Depending on the depth, the laser output and PMT (photomultiplier tube) sensitivity of the 2-photon microscope were changed.

Time-lapse imaging of the same view area in (c) at a 120 µm depth of the anesthetized mouse cortex was performed with a 2-photon microscope using a resonant scanner. The 10 times speed movie that was captured at 389.72 ms/frame and averaged at every 10 frames is shown in Supplementary Movie 5. Since the resonant scanner allows faster capture than the galvano scanner, the blinking of fluorescence spots is easier to recognize. It is thought that those fluorescence spots blinking represent the activity of excitatory neurons.

**(d)** Image shows the vertical cross section of the cortex (c). The length of 0-※ and 0-★ are 900 µm. Roman numerals on the right side of figure indicate the layer of the cortex. Bar indicates 100 µm.

**(e, f)** The fluorescence image of the same mouse as (a) which was taken by the HLC under the freely-moving condition at 57 days after surgical operation is shown in (e). Red square shows the imaging area by 2-photon microscopy, which is the same as the black square in (a). Bar indicates 100 µm. Each colored arrow roughly corresponds in position to the light spots in (f). The image of (e) is the result of the processing as the following. The fluorescence movie data taken by the HLC for 5 minutes at 50 ms/frame (20 fps) was deconvoluted and background subtracted, and all frames were merged to one image by a maximum intensity projection.

In (f), the fluorescence images of the anesthetized mouse were taken by 2-photon microscopy. Bar indicates 100 µm. The images at 1.00 sec/stack were captured to a depth of 600 µm at every 10 µm intervals (see for the Z axis reconstruction data in (d)). And then, images were superimposed every 100 µm by the maximum intensity projection of each of 10 slices, as shown “raw”. The blurring with a radius of 3.8 pixels at the light spot of that raw imaging data was performed as shown “blurred” by using a Gaussian filter of ImageJ. We performed this blurring procedure to match the resolution (9.62 µm/pixel) by the HLC whose view field in this experiment is 4.62 x 6.16 mm, 480 x 640 pixels to the resolution (1.24 µm/pixel) by 2-photon microscope. The difference in resolution between the HLC and 2-photon microscope is 7.7 times, therefore the radius of 3.8 pixels blurring was performed to obtain similar images.

As a result, during the long-term housing, neuronal activity could be visualized using the HLC and also by conventional 2-photon microscopy through the cranial window of the spacer apparatus.

**(g)** Furthermore, the fluorescence images in the GAD67-GCaMP6f mouse, GAD67-cre^s4,s5^ x cre dependent GCaMP6f (Jackson 024105)^13^, that were taken by 2-photon microscopy or the HLC in the similar way shown in (a) were compared. GAD67, glutamate decarboxylase, is a member of the group II decarboxylase family of proteins and are responsible for catalyzing the rate limiting step in the production of GABA (gamma-aminobutyric acid), and localizes in the inhibitory neurons that exist at all cortical layers sparsely. Therefore, the fluorescence from each inhibitory neuron was expected to be easier to distinguish than the case of excitatory neurons that exist high densely in the cortex. In (g), the left upper image was taken by 2-photon microscopy with x4 objective lens at the depth of 100 µm in the awake (unanesthetized) mouse. Blue broken line indicates the central of the brain, and the dura matter was removed at the right hemisphere but not at the left. The left lower image was taken in the anesthetized mouse by 2-photon microscopy with x20 objective lens at the red square area in the left upper image. The z-stacked image was merged images that were captured to a depth of 0-300 µm every 10 µm. Since the fluorescence does not blink in the GAD67-GCaMP6f mouse under the anesthetized condition, it is thought that the z-stacked image shows all existing fluorescence that is basal of GCaMP6f at 0-300 µm. Next, in the awake mouse at the same observation point (red square), the middle upper or lower image was taken by the HLC or 2-photon microscopy at the depth of 100 µm, respectively. The right upper image shows a maximized image of 5 minutes movie taken by the HLC, and that was subtracted with its first frame image. The right lower image shows the blurred image of the middle lower image with the radius of 2 pixels by Gaussian filter to obtain similar images taken by the HLC in the same reason in (f). Since the fluorescence blinks under the awake condition, the fluorescence intensity varies depending on the imaging timing. Although the HLC image is deep-focus, the 2-photon image is a section at a depth of 100 µm. Therefore, at least, the right lower image shows fewer light spots than the left lower image and its position should be consistent. And actually, the position of clear light spots in the right and middle lower image corresponds to the same position in the left lower image (e.g. yellow arrowhead), additionally, they are also corresponds in the right and middle upper image. The right upper image shows the blinking light spots exist, therefore, which mean that individual neuronal activity can be detected by the HLC as a discriminable fluorescence point.


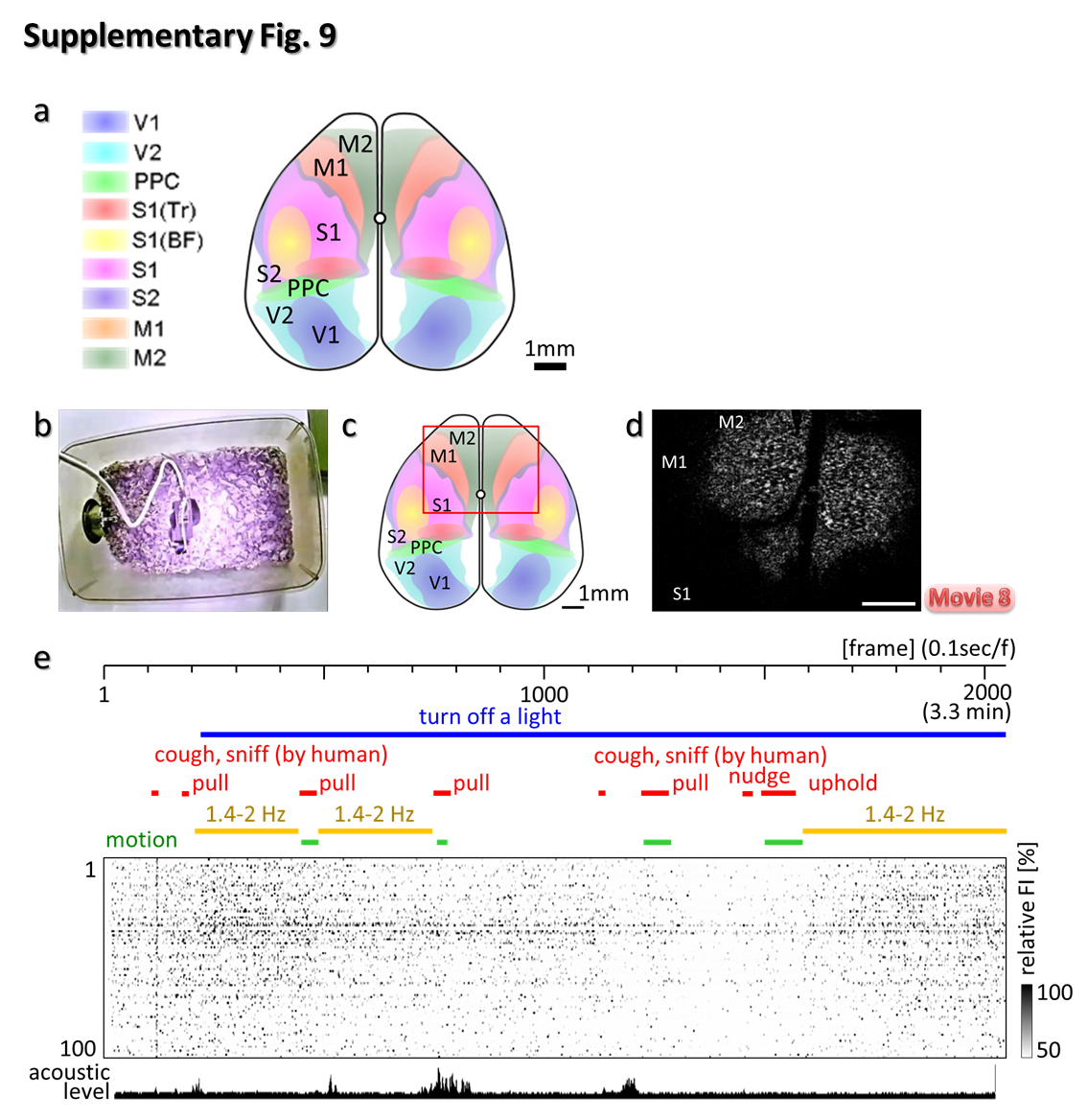


**Supplementary Fig. 9** Event related neuronal activity was observed in the frontal cortex in the freely moving condition

Ca^2+^ imaging was performed with the HLC in the freely moving CaMK2a-G-CaMP7 mouse in the square cage. The behavior of the mouse was taken with an IR web camera placed above the cage.

**(a)** The schematic image shows the cerebral cortical map which was partially modified from the map^37^ reconstructed from the serial coronal sections of the brain atlas^36^. Each abbreviation means V1/2, primary/secondary visual cortex; PPC (PtA), posterior parietal cortex (parietal association cortex); S1 (Tr/BF)/S2, primary (trunk/olfactory barrel field)/secondary somatosensory cortex; M1/2, primary/secondary motor cortex.

**(b)** The image shows the experimental setup.

**(c)** The schematic image of the HLC imaging area is shown (red square).

**(d)** The subtracted fluorescence image of the frontal cortex (c) in the CaMK2a-G-CaMP7 mouse taken by HLC under the freely moving condition (b) is shown. The raw movie (10 frame/sec) was subtracted by the averaged frame of the first 10 frames of the movie, and then the brightness and contrast was enhanced to observe neuronal activity in association with behavior as shown in (d, Supplementary Movie 8). Bar indicates 1 mm.

**(e)** 100 fluorescent spots of 4 pixels were picked up at random as representative ROIs from both hemispheres broadly based on the raw data. A temporal change of the FI in each ROI is shown by the raster plot. Several interventions were applied to the mouse during Ca^2+^ imaging (notation in red). The bottom graph shows an ambient acoustic level. The spacer of the HLC was shielded from light by the additional liquid rubber coat, and the changes in the surrounding lighting had no direct influence on the imaging result. The blue line indicates the period of dark environment, and the mouse behavior could still be observed by the IR web camera. Since the HLC has an IR cut filter (<650 nm), IR lighting also had no direct influence on the imaging result. The green lines indicate mouse motion.

When pulling, nudging, and upholding the mouse body were repeatedly conducted during Ca^2+^ imaging of the frontal cortex, neuronal activity changed in relation to these events. When the experimenter coughed near the mouse, transient and overall activity occurred in many ROIs. However, such a global reaction was not caused by a second cough, possibly due to acclimation. Broad slow synchronized wavelike activity propagated sometimes at the resting state during the periods indicated by yellow lines. Such a slow wave response was interrupted by a pull, and does not always occur during the resting period, which suggests that the phenomenon is not just noise. This slow oscillatory activity may be similar to the one observed during the inter trial periods^s6^.


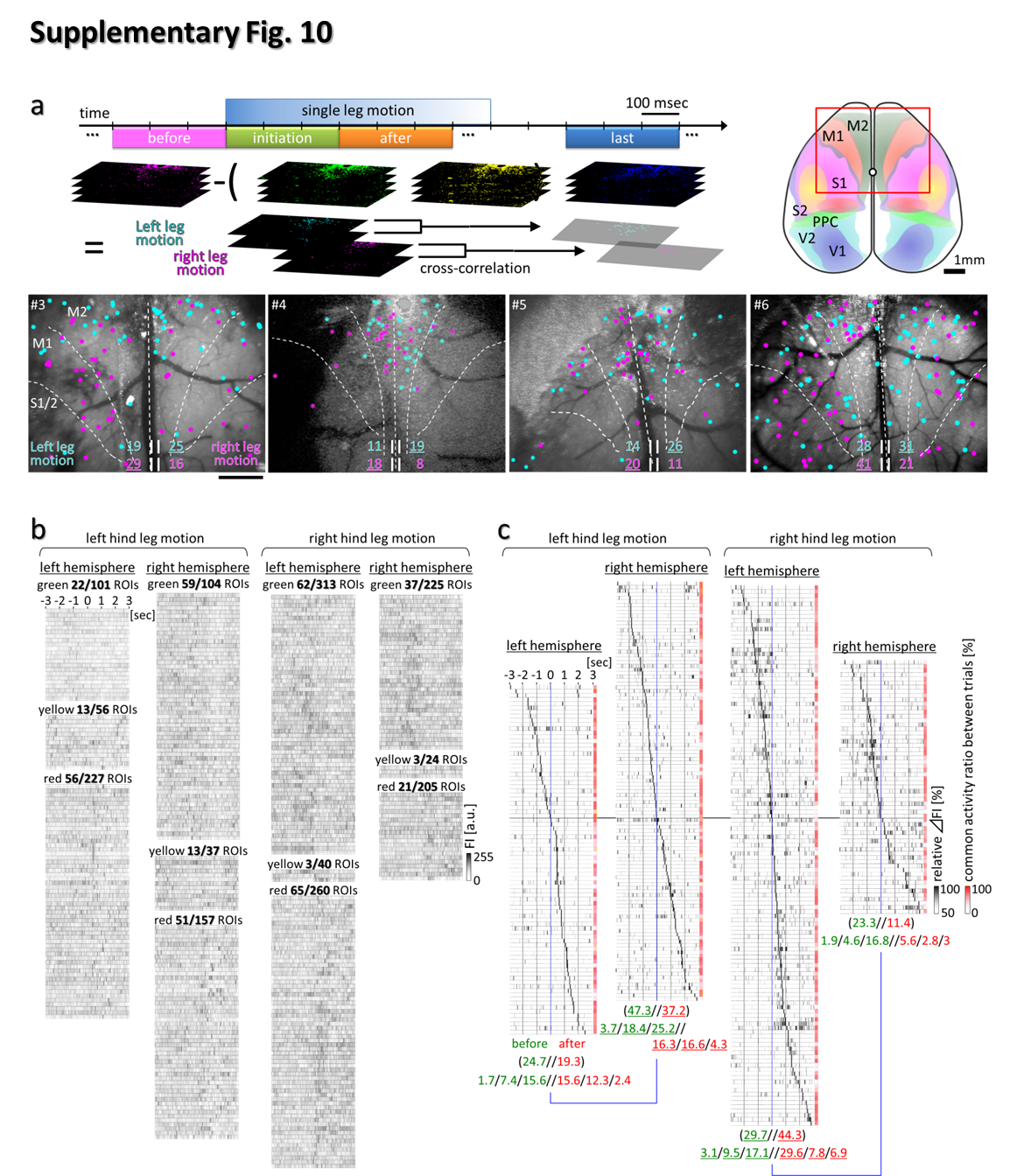


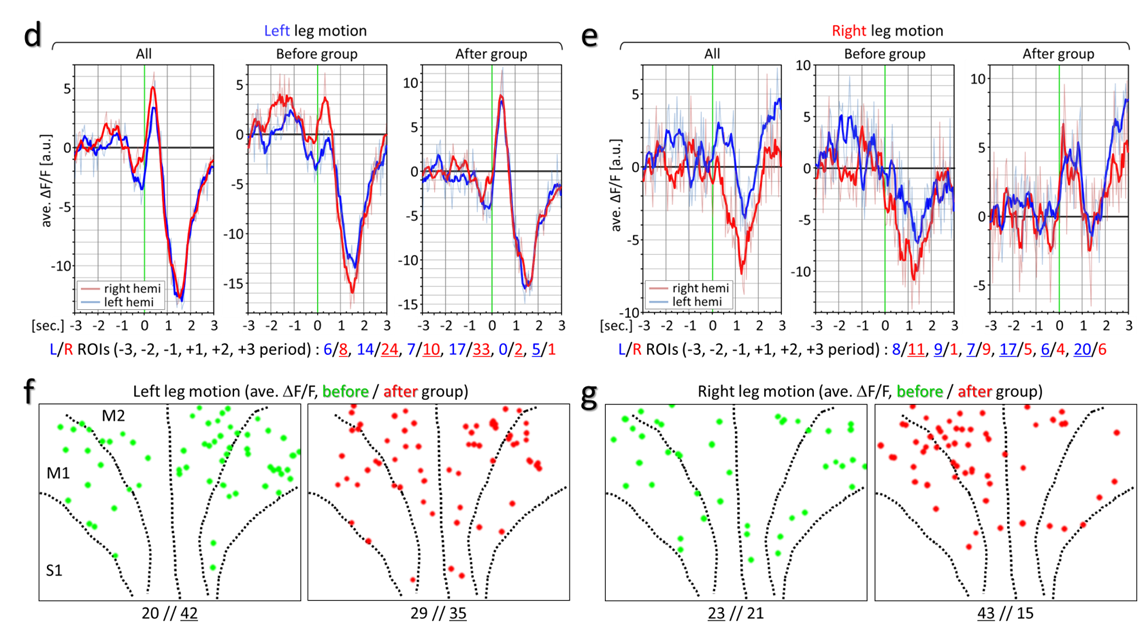


**Supplementary Fig. 10** Cross-correlation analysis and the relative ΔFI in the comprehensive quantitative analysis reveal the specific cell assemblies which represent a cascadic premotor activity during voluntary movement.

**(a)** The result shows the cross-correlation analysis of premotor activity. In the CaMK2a-G-CaMP7 mouse, 10 or 20 fps (frame per second) Ca^2+^ imaging was performed over the frontal cortex including the whole motor cortex by the restriction motion experiment and the HLC (Fig. 8a). The left schematic image indicates the process of the analysis method. The middle schematic image shows the imaging position by the HLC. The red square indicates the imaging area by the HLC, 5.0 x 6.5 mm. The activity positions of excitatory neurons during the 300 ms before the onset of the left or right hindleg motion were extracted in the process as described below. First, 4 periods for 300 ms during the left or right hindleg’s voluntary movement were categorized as “before”, “initiation”, “after” and “last”. For example, “before” or “initiation” was defined the period just before or after the onset of the motion, respectively. “After” was defined as the period just after “initiation”. “Last” was defined as the period from 200 ms just after the motion stopping. Next, fluorescence imaging data from each of the 4 periods was subtracted by the data of the one frame just before each period to reduce the background. Those subtracted imaging movie data were converted to a one frame image by maximization, and then, the subtraction between each period, max of “before” – max of [“initiation” + “after” + “last”], was performed for each ROI. The result was expected to represent only premotor activity. Finally, the image of two discrete trials accompanying the left or right hindleg’s motion in the same mouse is shown for comparison in the lower images of (a). The results of 4 mice are shown (mouse ID: #1 - #4). Bar indicates 1 mm. Cyan or magenta dots indicate the neurons activated before the left or right hindleg motion. For example in #1 mouse, 19 and 25 neurons of the left and right hemisphere, respectively, were activated before the left leg motion. And 29 and 16 neurons of the left and right hemisphere, respectively, were activated before the right leg motion. These neurons seem to be more localized to M2 than M1, and are rarely present in the other areas such as the somatosensory cortex, and its localization is biased toward the contralateral side over the motion side.

The results of (b, c) show a part of the comprehensive quantitative analysis of the premotor activity in Fig. 8.

**(b)** The temporal value changes of FI, derived from the raw 20 fps Ca^2+^ imaging data, at ROIs in the subtracted image of Fig. 8e with >5 % ΔF/F are presented by a raster plot. The numbers at the top of each raster plot mean [>5 % ΔF/F ROIs] / [all ROIs]. The ROIs were classified by color codes (green, yellow and red) according to the criteria described in the legend of Fig. 8e. The yellow ROIs are double positive for both green and red characters.

**(c)** The data shows the temporal raster plot of relative FI change rates of (b) (relative ΔFI [%]), and an averaged possibility of all kick events at each ROI (red boxes). The relative ΔFI was calculated so that a minimum or maximum value was set to 0 or 100 for each ROI in data of (b). Then, the 50-100 % relative ΔFI in each ROI was presented by the order of the peak point time. The possibility value was calculated based on whether the subtracted FI at each ROI in each kick event was positive (= 1) or negative (= 0). The averaged possibility values at each ROI in all kick events are indicated by red boxes at the right end of the raster plot line. The sum of possible values is indicated by the number under each raster plot. Green or red colored numbers mean the sum before or after the initiation of the motion. The bottom numbers mean the sums within each time period (-3 to -2 / -2 to -1 / -1 to 0 // 0 to 1 / 1 to 2 / 2 to 3 [sec]). Underline means the larger numbers compared to those on the other side of the hemisphere. These results also indicate the tendency that many active ROIs exist in the contralateral hemispheres from the motion side similar to the result in (a).

Next, an overall qualitative analysis was performed to eliminate the concern that accidentally large but rare activities might make unduly great contributions by maximization analysis.

**(d, e)** Left graphs indicate the averaged total ΔF/F of all ROIs (b) during all left or right kick events. ΔF/F was measured from raw data of each event at the ROIs (b) excluding those of the <50 % possibility and the somatosensory area. Then, the ΔF/F of 11 or 10 trials were all averaged for each hemisphere. The middle or right graphs indicate the averaged total ΔF/F of the specific cell assembly whose peak of ΔF/F appeared before or after the initiation of the leg movement (abbreviated as “before group”, “after group”). Bold red and blue lines indicate the mean of FI of each 5 frames. The bottom numbers indicate the numbers of the ROIs in the left or right hemisphere within each time period (-3 to -2 / -2 to -1 / -1 to 0 // 0 to 1 / 1 to 2 / 2 to 3 [sec]). The underlines mean that the number is larger than on the other side. As a result, the lateralized activity was observed, especially at a period from -2 to +2 sec, just before and after the initiation of the motion in left graphs.

These three differential activities are also basically observed in the middle graphs representing the result of the “before group” cell assembly. Surprisingly, however, in the “after group” cell assemblies (right graphs), there was almost no difference in the left-right activities at periods from 0 to +2 sec. In contrast, the “before group” cell assemblies (middle graphs) indicate the different left-right activities before and after the onset of the motion.

In conclusion, our data suggest that motor planning for the left or right hindleg specific movements has been already completed by the lateralized activation of the “before group” cell assembly before the onset of the movement which lasts even after the initiation of the movements.

**(f, g)** The “before group“ and “after group“ cell assemblies of (d, e) were plotted on the schematic diagram of the motor cortical area whose regional compartments are the same as the image of Fig. 8d. The bottom numbers mean the numbers of the ROIs in the left or right hemisphere. The underlines mean that the number is larger than on the other side. As a result, the tendency of the lateralized distribution also appears in the overall qualitative analysis similarly to visual judgment, cross-correlation analysis, and comprehensive quantitative analysis.

In summary, lateralized premotor activities seem to start from M2 on each opposite side of the hemisphere, and the activities propagate to M1 and the somatosensory region. The trends of our results are largely consistent among all the different analyses that were conducted in this paper. The result that the volitional movement originates from M2 is consistent with previous reports and supports them^18,s7-s10^. The pre- and post-motor activities in M1 have been occasionally pointed out to be non-lateralized^18,s11,s12^, and even ipsilateral^s13,s14^. In fact, the “after group” cell assemblies of (d, e) show bilateral non-lateralized activity during the period from 0 to +2 sec. However, the “before group“ cell assemblies of (d, e) represent differential activity, and the 2D distribution study in Fig. 8f showed that the pre- and post-motor activities in M1 are also lateralized similarly as well as in M2. Therefore, we conclude that M1 activity is lateralized.

The lateralized premotor activity at just before the initiation of the motion (-0.5 to 0 sec) probably represents the start of a direct motor command in the neurons projecting to motor neurons controlling the muscle, and the period includes a point of no return in vetoing the motion (around -0.2 sec)^s15^. The direct activity in M1 occurs 50 to 80 ms before muscle movement^s16^.

**Supplementary Movie 1** Examples of imaging the freely moving mouse wearing the HLC and spacer apparatus (Fig. 1c) under various conditions.

**Supplementary Movie 2** Ca^2+^ imaging by the HLC over the occipital cortex of an awake CaMK2a-G-CaMP7 mouse (Fig. 1d).

In the movie, (a) shows a movie where the background of the raw imaging movie (10 frame/sec) was subtracted by using ImageJ with a sliding paraboloid (curvature radius is 200 pixels), and was deconvoluted. (b) is one frame of the movie (a). (c) is the magnified movie of the inset shown in (b). (d) is the same as (b). (e) was made by subtracting the FI of each pixel with the average of the FI of the same pixel during the first 10 times.

**Supplementary Movie 3** The procedure for counting the number of blinking spots in the view field of Fig. 1d.

In the movie, (a) shows the maximized movie that 8 movies of 1 min were merged by taking the maximum FI for each pixel. (b), (c) show the same image of Supplementary Fig. 4a, b, respectively.

**Supplementary Movie 4** x10 speed Ca^2+^ imaging by the HLC on the 3D cultured G-CaMP6-Hela with histamine administration (Supplementary Fig. 7a, raw fluorescence image is shown in Fig. 4a).

**Supplementary Movie 5** x20 speed Ca^2+^ imaging with a 2-photon microscope over the motor cortex of the anesthetized CaMK2a-G-CaMP7 mouse (Supplementary Fig. 8c).

**Supplementary Movie 6** x100 speed Ca^2+^ imaging by the HLC on the somatosensory cortical area of the anesthetized Thy1-G-CaMP7 mouse (Fig. 4d, raw fluorescence time-lapse image is shown in Supplementary Fig. 7c).

**Supplementary Movie 7** Magnified Ca^2+^ imaging by using the HLC with short spacer in the freely moving mouse.

Ca^2+^ imaging of the visual cortex of the CaMK2a-G-CaMP7 mouse was performed by the HLC with the short spacer under the freely moving condition. The 20-fps movie was magnified by the optical zoom (x 2 in comparison with Supplementary Fig. 4) and the digital zoom (x 4) is shown (Fig. 5).

**Supplementary Movie 8** The Ca^2+^ imaging by the HLC of the frontal cortex in the freely moving CaMK2a-G-CaMP7 mouse (Supplementary Fig. 9b, d).

The left and right movies show a behavior movie of the mouse and the subtracted and averaged Ca^2+^ imaging movie, respectively.

**Supplementary Movie 9** x1/10 Ca^2+^ imaging by the HLC on the motor cortical area of the awake CaMK2a-G-CaMP7 mouse (Fig. 8b).

Left/ middle/ right movies show a behavior movie of the mouse, the raw Ca^2+^ imaging movie, and the marked raw Ca^2+^ imaging movie, respectively. In the right movie, the magenta or green dots mean the neuronal activities which start during the before (-4 to 0 sec) or after (0 to +4 sec) the initiation of the motion. Several arrows indicate the dots and its corresponding positions.

**Supplementary Movie 10** Ca^2+^ imaging by the single and dual HLC under the freely moving condition.

Real-time Ca^2+^ imaging of the awake CaMK2a-G-CaMP7 mouse by the single HLC of the frontal cortical area (Supplementary Fig. 3d) or by the dual HLC of the frontal and occipital cortical area (Supplementary Fig. 2d) were performed. The movie shows 30-fps captures of the video images displayed on the PC monitor. Judging from the orientation position of the blood vessel, the vibration induced by mouse’s movements is not recognized and the view field does not change. Also, even if the two HLCs are used, the movement of the mouse is not impaired.

**Supplementary References**

s1. Tribromoethanol (Avertin). *Cold Spring Harbor Protocols* **2006,** pdb.rec701 (2006).

s2. Kawai, S., Takagi, Y., Kaneko, S. & Kurosawa T. Effect of three types of mixed anesthetic agents alternate to ketamine in mice. *Exp Anim* **60,** 481-487 (2011).

s3. Shibuki, K. et al. Dynamic imaging of somatosensory cortical activity in the rat visualized by flavoprotein autofluorescence. *J Physiol* **549,** 919-927 (2003).

s4. Higo, S., Akashi, K., Sakimura, K. & Tamamaki, N. Subtypes of GABAergic neurons project axons in the neocortex. *Front Neuroanat* **3**, 25 (2009).

s5. Wu, S. et al. Tangential migration and proliferation of intermediate progenitors of GABAergic neurons in the mouse telencephalon. *Development* **138**, 2499-2509 (2011).

s6. Zagha, E., Ge, X. & McCormick, D.A. Competing neural ensembles in motor cortex gate goal-directed motor output. *Neuron* **88,** 565-577 (2015).

s7. Komiyama, T. et al. Learning-related fine-scale specificity imaged in motor cortex circuits of behaving mice. *Nature* **464,** 1182-1186 (2010).

s8. Hira, R. et al. Spatiotemporal dynamics of functional clusters of neurons in the mouse motor cortex during a voluntary movement. *J Neurosci* **33,** 1377-1390 (2013).

s9. Li, N., Chen, T.W., Guo, Z.V., Gerfen, C.R. & Svoboda, K. A motor cortex circuit for motor planning and movement. *Nature* **519,** 51-56 (2015).

s10. Makino, H. et al. Transformation of cortex-wide emergent properties during motor learning. *Neuron* **94**, 880-890 (2017).

s11. Donchin, O. et al. Single-unit activity related to bimanual arm movements in the primary and supplementary motor cortices. *J Neurophysiol* **88,** 3498-3517 (2002).

s12. Cisek, P., Crammond, D.J. & Kalaska, J.F. Neural activity in primary motor and dorsal premotor cortex in reaching tasks with the contralateral versus ipsilateral arm. *J Neurophysiol* **89,** 922-942 (2003).

s13. Verstynen, T., Diedrichsen, J., Albert, N., Aparichio, P. & Ivry, R.B. Ipsilateral motor cortex activity during unimanual hand movements relates to task complexity. *J Neurophysiol* **93,** 1209-1222 (2005).

s14. Derosiere, G. et al. Similar scaling of contralateral and ipsilateral cortical responses during graded unimanual force generation. *Neuroimage* **85,** 471-477 (2014).

s15. Schultze-Kraft, M. et al. The point of no return in vetoing self-initiated movements. *Proc Natl Acad Sci U S A* **113,** 1080-1085 (2016).

s16. Kurata, K. & Tanji, J. Premotor cortex neurons in macaques: activity before distal and proximal forelimb movements. *J Neurosci* **6,** 403-411 (1986).
